# Assessing a megadiverse but poorly known community of fishes in a tropical mangrove estuary through environmental DNA (eDNA) metabarcoding

**DOI:** 10.1038/s41598-022-19954-3

**Published:** 2022-09-29

**Authors:** Danial Hariz Zainal Abidin, Siti Azizah Mohd. Nor, Sébastien Lavoué, Masazurah A. Rahim, Noor Adelyna Mohammed Akib

**Affiliations:** 1 Centre for Global Sustainability Studies (CGSS), Level 5, Hamzah Sendut Library, Universiti Sains Malaysia, 11800 Penang, Malaysia; 2 School of Biological Sciences, Universiti Sains Malaysia, 11800 Penang, Malaysia; 3 Institute of Marine Biotechnology, Universiti Malaysia Terengganu, 21030 Kuala Terengganu, Terengganu Malaysia; 4 Fisheries Research Institute, 11960 Batu Maung, Penang Malaysia

**Keywords:** Next-generation sequencing, Biodiversity, Conservation biology

## Abstract

Biodiversity surveys are crucial for monitoring the status of threatened aquatic ecosystems, such as tropical estuaries and mangroves. Conventional monitoring methods are intrusive, time-consuming, substantially expensive, and often provide only rough estimates in complex habitats. An advanced monitoring approach, environmental DNA (eDNA) metabarcoding, is promising, although only few applications in tropical mangrove estuaries have been reported. In this study, we explore the advantages and limitations of an eDNA metabarcoding survey on the fish community of the Merbok Estuary (Peninsular Malaysia). COI and 12S eDNA metabarcoding assays collectively detected 178 species from 127 genera, 68 families, and 25 orders. Using this approach, significantly more species have been detected in the Merbok Estuary over the past decade (2010–2019) than in conventional surveys, including several species of conservation importance. However, we highlight three limitations: (1) in the absence of a comprehensive reference database the identities of several species are unresolved; (2) some of the previously documented specimen-based diversity was not captured by the current method, perhaps as a consequence of PCR primer specificity, and (3) the detection of non-resident species—stenohaline freshwater taxa (e.g., cyprinids, channids, osphronemids) and marine coral reef taxa (e.g., holocentrids, some syngnathids and sharks), not known to frequent estuaries, leading to the supposition that their DNA have drifted into the estuary through water movements. The community analysis revealed that fish diversity along the Merbok Estuary is not homogenous, with the upstream more diverse than further downstream. This could be due to the different landscapes or degree of anthropogenic influences along the estuary. In summary, we demonstrated the practicality of eDNA metabarcoding in assessing fish community and structure within a complex and rich tropical environment within a short sampling period. However, some limitations need to be considered and addressed to fully exploit the efficacy of this approach.

## Introduction

Estuaries associated with mangrove forests are transitional ecosystems between freshwater and marine environments, serving essential ecological functions including protective, feeding, spawning, and rearing habitats for a diverse array of aquatic organisms^1^. Mangroves provide critical natural services such as coastline protection, nutrient synthesis, and fishery resources, which are essential for sustaining local communities’ socioeconomic livelihoods^2^. Regrettably, such vital human-nature relationships are jeopardized by habitat degradation, pollution, and overfishing^3^. Well-protected tropical estuaries, particularly those comprising mangrove habitats, support diverse and complex biological communities that include euryhaline resident species as well as frequenters, including numerous marine species, that rely on this ecosystem for food, shelter, breeding, or nursing^4^. Assessing the ecosystem’s diversity and community structure is critical for evaluating its health and key to successfully protect its biodiversity^5,6^. Thus, inventories and long-term biodiversity surveillance of native communities in estuarine mangrove ecosystems are required to ensure stability and resilience against anthropogenic disturbance, monitoring alien species invasion, and to prevent the loss of native, sometimes endemic, species^1,6,7^. Long-term monitoring efforts aid in identifying which species are most vulnerable to environmental stressors, determining new threats, and revealing the interconnectedness among species in an ecosystem^8^. Conventional methods are, however, time-consuming, often cost-prohibitive and requires expertise in traditional taxonomy^9^. Consequently, biodiversity in many mangrove habitats, notably those in Southeast Asia, is still not adequately monitored, impeding their management and conservation.

**Figure 1:**
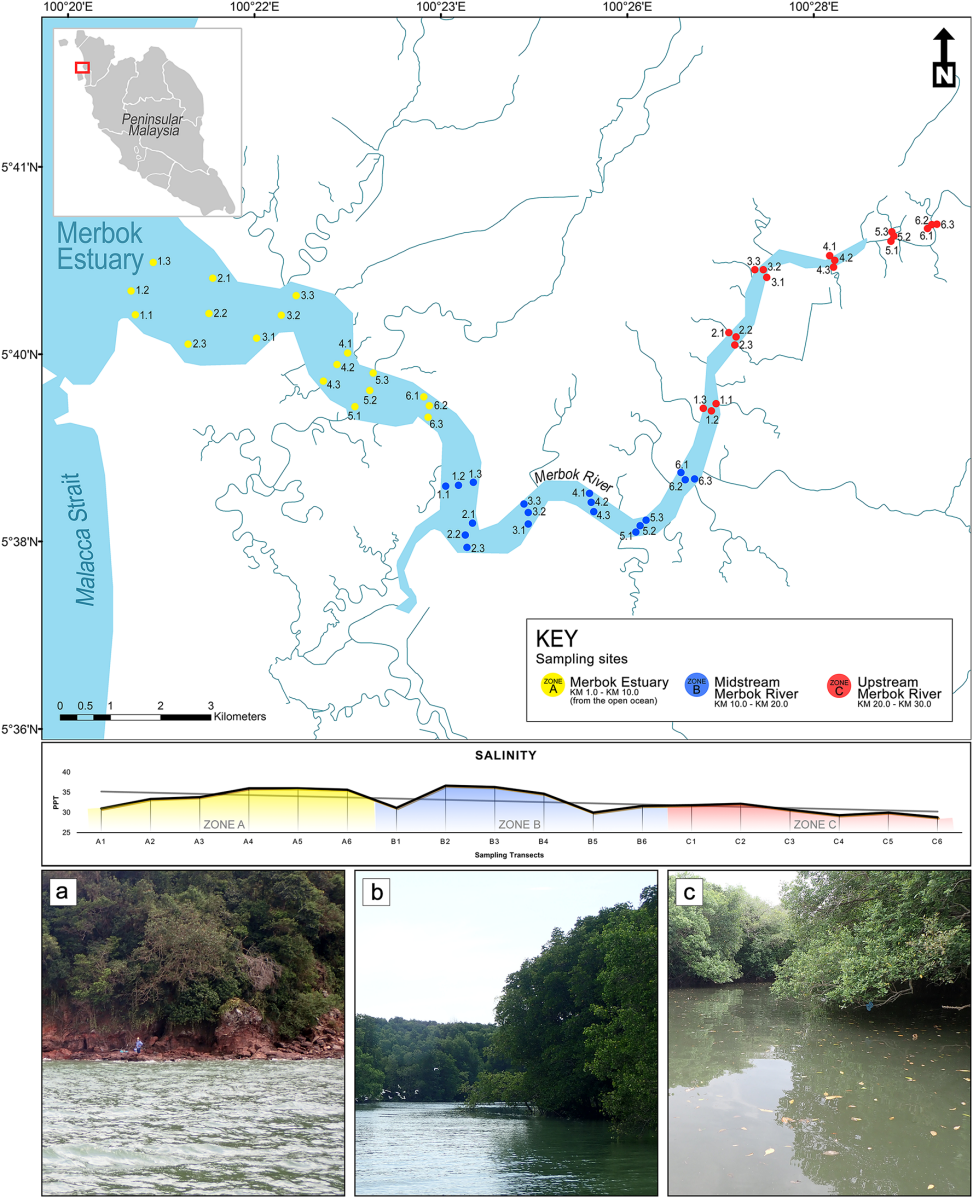
Location of sampling sites across the Merbok Estuary. Inset map shows the location of the study area within Peninsular Malaysia. The diagram shows the salinity measurements at the sampling sites, categorised by colour - shaded yellow: Zone A; blue: Zone B; and red: Zone C. Photographs of the representative sites within the three zones: (**a**) site A1.1, (**b**) site B3.2, (**c**) site C6.3. Map is generated using ArcMap 10.8 (http://www.esri.com) and edited in Adobe Photoshop CC 2019 (https://www.adobe.com/products/photoshop.html).

Located within the Sundaland biodiversity hotspot^10^, Malaysia is home to astonishing levels of diversity and endemism. In particular^11^, reported the presence of more than 1400 marine and brackish species in coastal Malaysian waters, a significant proportion either residing or frequenting mangrove ecosystems. The Merbok Estuary, located in northwest Peninsular Malaysia (Fig. 1), is not only one of the region’s largest remaining patches of mangrove forest (~ 40,000 ha)^12^, it is also recognised as the World’s most diverse ecosystem for mangrove species in terms of species richness per unit area^13^. In 1951, the Merbok Estuary was gazetted as a permanent forest reserve known as the Sungai Merbok Mangrove Forest Reserve. In spite of its value and critical role in providing vital ecosystem services and livelihoods to the local community, few biodiversity inventories have been conducted in Merbok Estuary^14–16^. Recent ichthyodiversity surveys in Merbok Estuary using three different approaches (morphological and DNA barcoding on adults, and metabarcoding of larvae) revealed the presence of about 180 species^7,12,17^. Although these studies discovered a large number of species, their respective list of species only partially overlapped to each other, indicating limitations in each approach and incomplete taxonomic coverage. Thus, there is a need to explore alternative monitoring approaches for building an exhaustive species diversity list^18,19^.

Environmental DNA (eDNA) metabarcoding is recognised as a revolutionary method to effectively conduct multi-taxa inventory surveys using bulk DNA extracted from environmental samples (e.g. water, soil)^20,21^. It overcomes the shortcomings of conventional survey methods to efficiently characterise fish assemblages in aquatic habitats as have been reported in estuarine^22–24^, marine^25,26^, and freshwater ecosystems^27,28^. The use of eDNA as a powerful monitoring instrument is increasingly acknowledged as it reveals greater diversity at a lower cost compared to conventional surveying methods^29–32^. The eDNA metabarcoding method is said to outperform conventional surveys in taxa detection (i.e., identification of novel species and conservation targets) and determination of the local community composition^29,32^. This approach has also been employed to address other objectives, including detection of invasive species^33,34^, identification of cryptic species^35,36^, understanding spawning ecology^37,38^, and in assessing ecosystem health and dynamics^39^.

In this study, we utilised the eDNA metabarcoding method to estimate fish diversity in the Merbok Estuary. Through this, we aim to augment the Merbok Estuary previous checklists assembled through traditional surveys towards a complete species database. We also evaluated the complementarity of two eDNA metabarcoding assays (each based on a different mitochondrial marker, the cytochrome oxidase I [COI] and 12S rRNA [12S]) to characterise the diversity patterns and composition of fish communities in this ecologically and biologically diverse landscapes of Merbok Estuary. Our study demonstrated the potential of eDNA metabarcoding as an efficient tool in inventorying the fish diversity of this globally important mangrove hotspot.

## Results

### eDNA-based fish detection in Merbok Estuary

A total of 12,958,643 and 10,757,026 raw amplicon reads were generated from the COI and 12S assays, respectively (Supplementary Table 4). The mean numbers of filtered reads (after post-quality processing and chimera removal) were 184,017 and 140,118 per sample for COI and 12S assays, respectively. Of these, the COI metabarcoding assay identified 8,332 Molecular Operational Taxonomic Units (MOTUs) whereas the 12S assay identified 859 MOTUs. The COI primer pair amplified taxa from multiple animal phyla (e.g., Insecta, Gastropoda, Aves, Mammalia, Chondrichthyes, and Actinopterygii), whereas the 12S primer pair was more vertebrate-specific in amplifying primarily Actinopterygii, Amphibia, Aves, and Mammalia, along with a small percentage of diatoms. The COI assay detected almost ten times more MOTUs compared to the 12S assay, with many of the COI MOTUs assigned to non-fish taxa. 98.51% of the 12S reads were assigned to fish taxa whereas only a mere 3.90% of the COI reads were assigned to fish taxa (Supplementary Table 4). The final taxonomic assignment after multiple filtering layers resulted in a total of 71 Actinopterygii and seven Chondrichthyes MOTUs identified from COI metabarcoding assay and 235 Actinopterygii MOTUs from 12S metabarcoding assay. Most fish species identified in both assays are represented by several MOTUs. Only few species are represented by a single MOTU.

**Figure 2:**
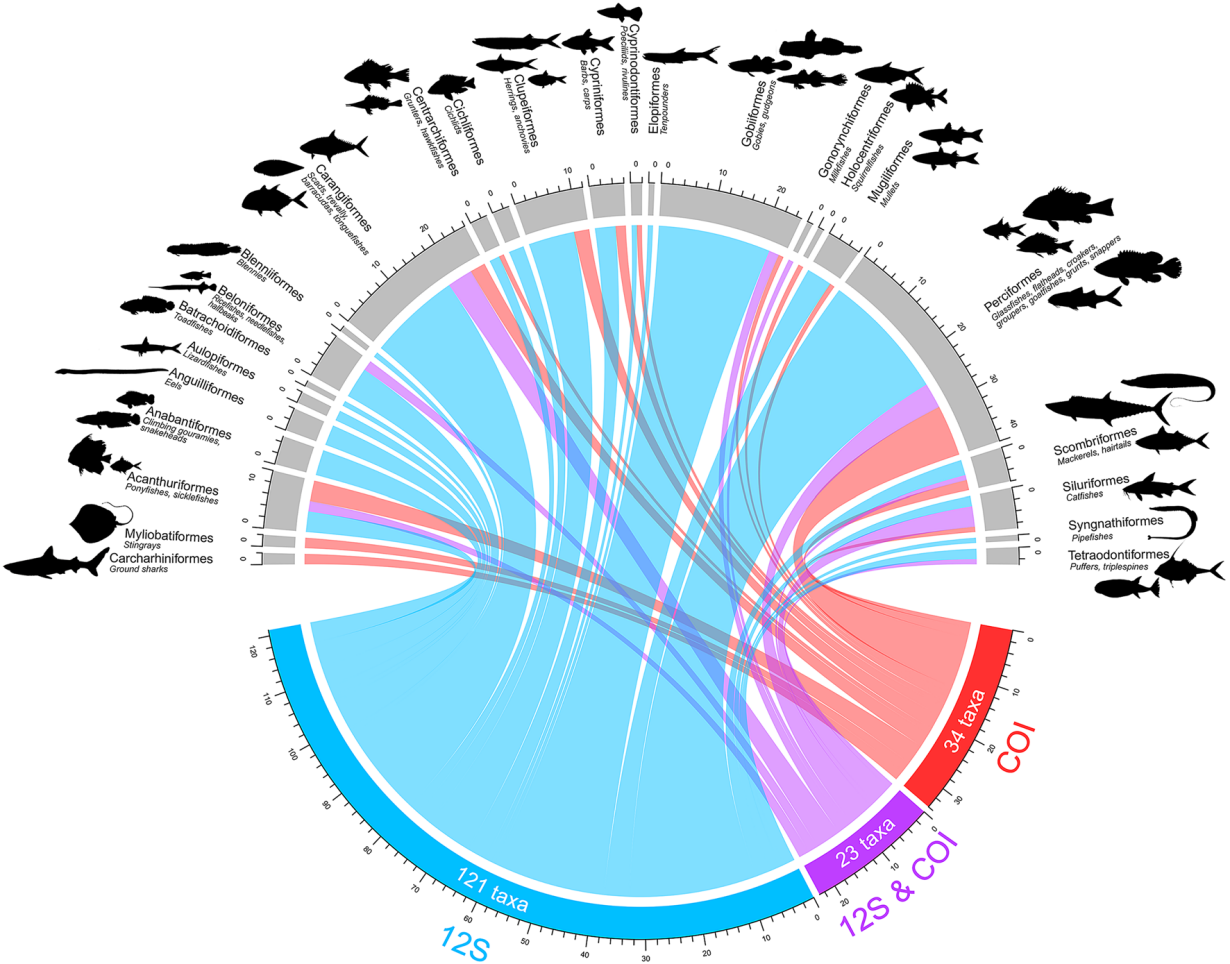
Actinopterygian and elasmobranch taxa detected from eDNA by the COI and 12S metabarcoding assays. *Circlize* plot showing assays being mapped to the 25 orders detected by eDNA metabarcoding. The purple ribbons represent overlapped detections from both COI and 12S metabarcoding assays.

Collectively, the COI and 12S metabarcoding assays detected 178 fish species belonging to 127 genera, 68 families, and 25 orders (Supplementary Table 2). Overlapped species detections and cross-amplification of taxonomic groups between both assays are visualised in the *circlize* plot^40^ (Fig. 2). Of these, 174 were bony fish species (class: Actinopterygii) and four were elasmobranch species (class: Chondrichthyes). The most speciose orders of bony fishes were Perciformes (with 40 species representing 22.5% of the total number of species detected), followed by Carangiformes (26 species, 14.6%), and Gobiiformes (25 species, 14%). Among these, the predominant families include Gobiidae (gobies; 18 species), Carangidae (jacks and pompanos; 13 species), Serranidae (groupers and sea basses; 9 species), and Sciaenidae (croakers; 8 species). The four elasmobranch species belong to three families, Carcharhinidae (requiem sharks; 2 species), Dasyatidae (whiptail stingrays; 1 species), and Gymnuridae (butterfly rays; 1 species). We detected seven fish species that are listed as either ‘Endangered’ (EN) or ‘Near Threatened’ (NT) in the IUCN Red List of Threatened Species (IUCN, 2020): blacktail reef shark (*Carcharhinus amblyrhynchos*, EN), blacktip reef shark (*Carcharhinus melanopterus*, NT), scaly whipray (*Brevitrygon walga*, NT), long-tailed butterfly ray (*Gymnura poecilura*, NT), Indonesian shortfin eel (*Anguilla bicolor*, NT), Bombay-duck (*Harpadon nehereus*, NT), and narrow-barred Spanish mackerel (*Scomberomorus commerson*, NT). Two of the detected species were introduced and classified as invasive species: Nile tilapia (*Oreochromis niloticus*) and the guppy (*Poecilia vivipara*).

### Comparison of eDNA metabarcoding detections with previous specimen-capture records

**Figure 3:**
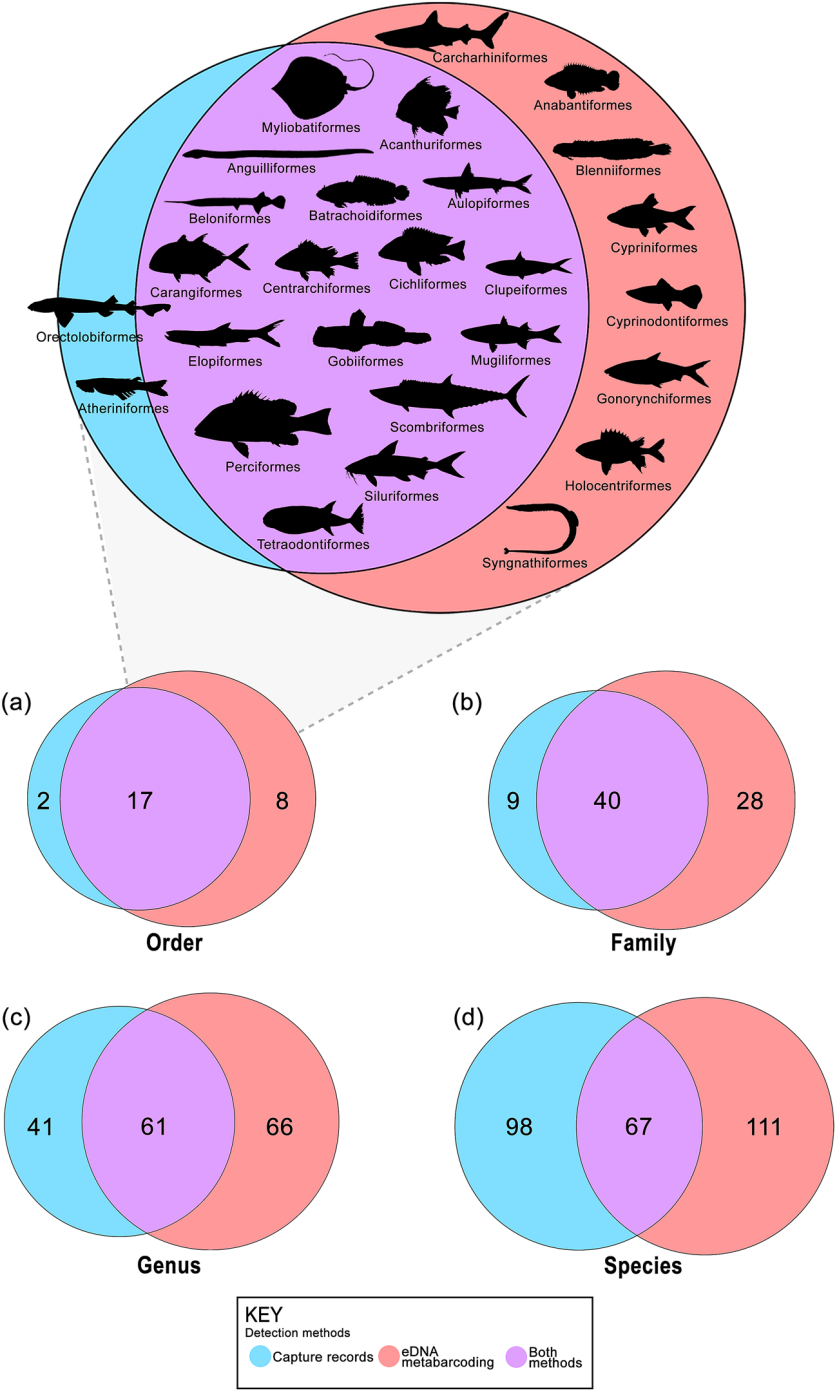
Venn diagram showing the number of taxa recovered from the local capture records (blue), eDNA metabarcoding assays (red), and both methods (purple) at different taxonomic ranks: (**a**) order, (**b**) family, (**c**) genus, and (**d**) species. Blown-up diagram at the top showing the orders detected by both methods.

Sampling campaigns conducted in Merbok Estuary during the last decade (2010-2019) recorded a total of 165 species from 102 genera, 49 families, and 19 orders^7,12,16^ whereas our eDNA metabarcoding assessment conducted in the same area recorded a total of 178 species (Fig. 3; Supplementary Table 2; Supplementary Table 3). This eDNA metabarcoding study captured far more species than any single previous traditional survey, overall resulting in a 40% increase in species diversity.

Overlapping taxa detected from both methods at different taxonomic ranks is shown in Fig. 3. Compared to the specimen-capture records, about 90% (17 orders), 82% (40 families), 60% (61 genera), and 41% (67 species) were also detected by eDNA metabarcoding assays at ordinal, familial, generic, and specific levels, respectively. The most speciose orders detected by both methods were Perciformes, Carangiformes, and Gobiiformes. The eDNA metabarcoding detected an additional eight orders: Carcharhiniformes (ground sharks), Anabantiformes (gouramies), Blenniiformes (blennies), Cypriniformes (carps and minnows), Cyprinodontiformes (killifish), Gonorynchiformes (milkfish), Holocentriformes (squirrelfishes and soldierfishes), and Syngnathiformes (trumpetfishes). In addition, the metabarcoding assays unexpectedly detected stenohaline freshwater species belonging to the families Cyprinidae (e.g., *Barbodes binotatus*, *Mystacoleucus obtusirostris*, *Tor tambra*, *Rasbora pauciperforata*), Channidae (*Channa limbata* and *C. striata*), and Osphronemidae (*Trichogaster lalius* and *Trichopodus trichopterus*) (Supplementary Table 2). Conversely, nine families recorded by conventional surveys were not detected by eDNA metabarcoding, namely the families Hemiscylliidae (longtailed carpet sharks), Dussumieriidae (round herrings), Hemiramphidae (halfbeaks), Phallostethidae (priapium fish), Paralichthyidae (sand flounders), Bothidae (lefteye flounders), Lethrinidae (emperor breams), Drepanidae (sicklefishes), and Stromatidae (butterfish).

### Diversity patterns and composition

**Figure 4:**
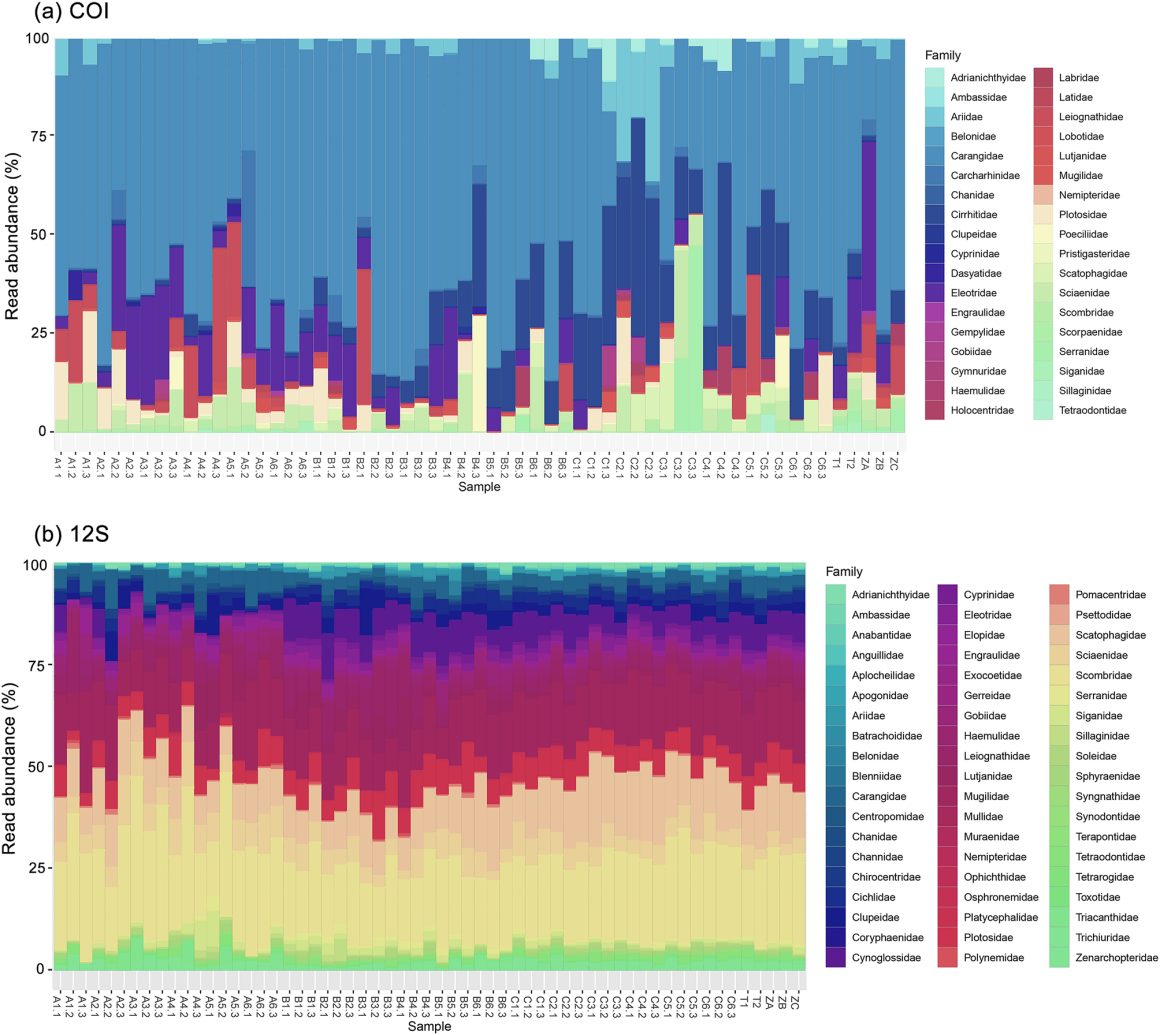
Barplots showing relative read abundance in all samples per fish family; (**a**) COI assay and (**b**) 12S assay.

The MOTU richness recovered from the two metabarcoding assays appeared to vary across samples and designated zones (Fig. 4). The COI assay detected many brackish and marine fishes belonging to the orders Carangiformes, Gobiiformes, Centrarchiformes, and Perciformes. The zones closer to the river mouth (Zones A and B) were dominated by marine families such as Carangidae, Cirrhitidae, Eleotridae, Sciaenidae, and Plotosidae, which are associated with pelagic habitats or sandy substrates (Supplementary Fig. 1). Interestingly, we detected the presence of the blacktail reef shark, *Carcharhinus amblyrhynchos* (identity = 100%, E-value = 0.021), a marine species, with the highest read abundance recorded from site A5.2 from the COI assay. The 12S metabarcoding assay however, revealed richer species diversity, in particular from the families Scombridae, Mugilidae, Scatophagidae, Sciaenidae, and Plotosidae (Supplementary Fig. 1). Notwithstanding, the MOTU analysis of both assays on the five pooled samples (samples TA, TB, TC, T1, and T2) did not detect any additional species.

**Figure 5:**
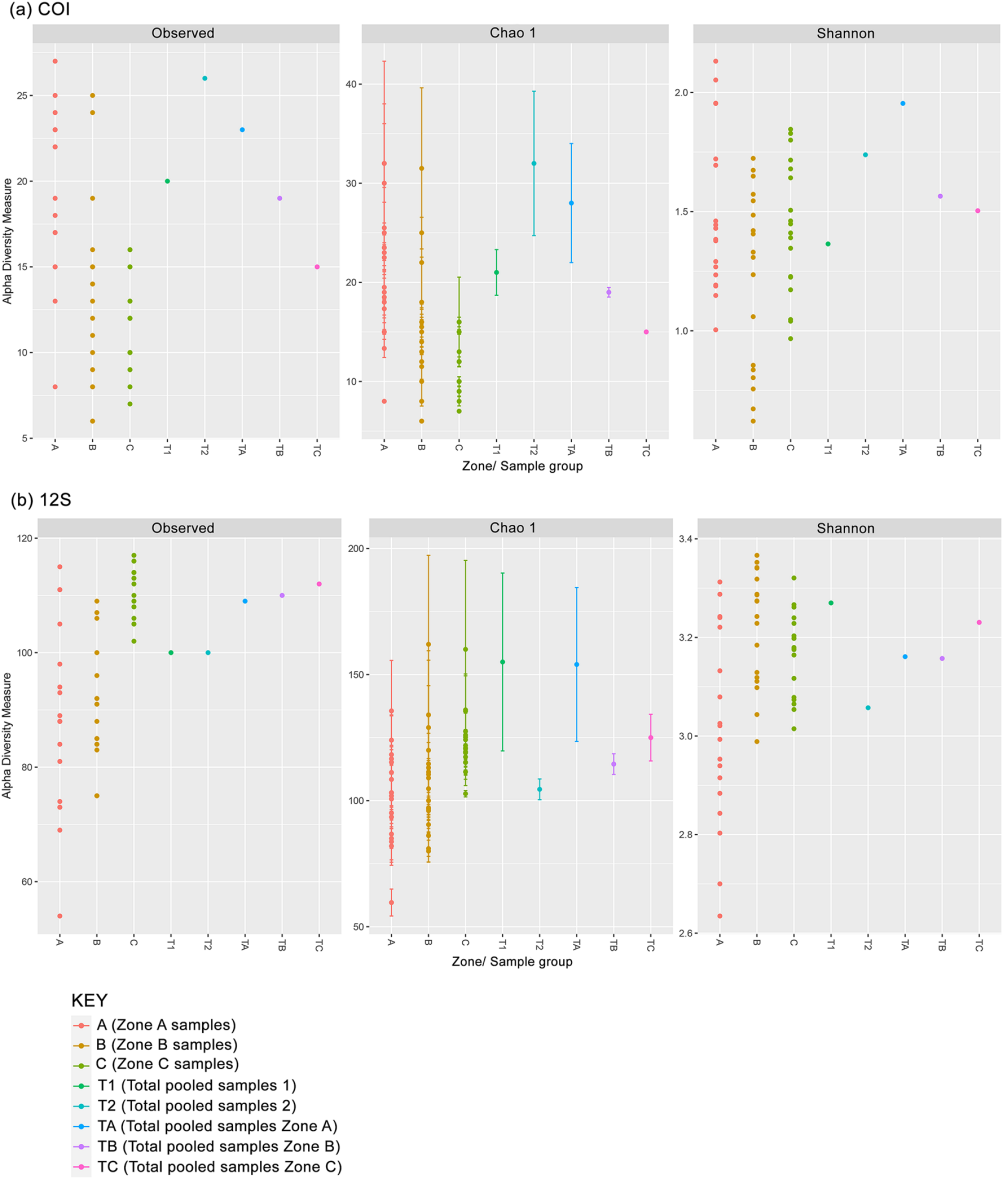
Alpha (α) diversity plots based on Observed, Chao1, and Shannon estimators grouped by zones and sample pools: (**a**) COI assay and (**b**) 12S assay.

**Figure 6:**
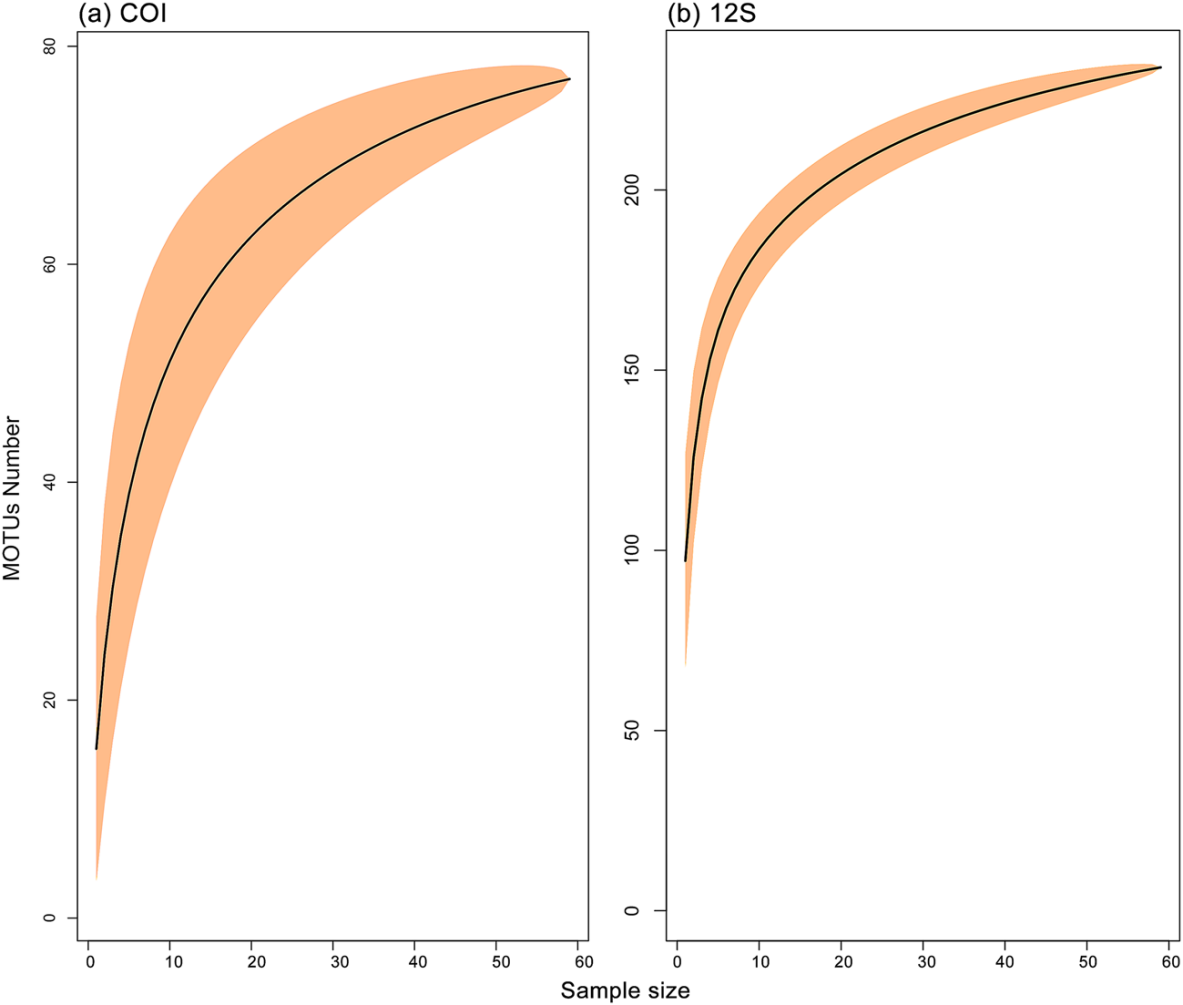
MOTU accumulation curves representing the number of MOTU identified in all samples analysed by eDNA metabarcoding assays: (**a**) COI assay and (**b**) 12S assay. The light-shaded area equates to the 95% confidence interval.

**Figure 7:**
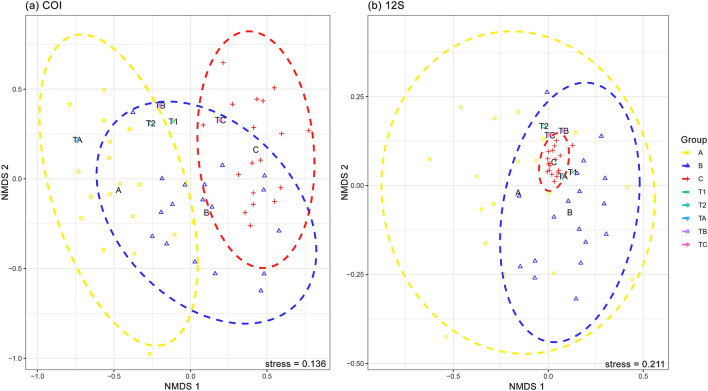
Nonmetric multidimensional scaling (NMDS) ordination of fish community in Merbok Estuary using the Bray–Curtis coefficient for both metabarcoding assays: (**a**) COI assay and (**b**) 12S assay. Different symbols denote individual samples analysed and the community clusterings are colour-coded (see legend). The ordination stress value is indicated at the bottom of each plot.

The α-diversity analysis based on the indices of richness (observed MOTUs and Chao1) and diversity (Shannon) showed varied patterns among the designated zones for both COI and 12S assays after rarefaction (Fig. 5). The coastal area of the Merbok Estuary (Zone A) harboured the highest MOTU richness based on COI whereas the 12S estimated higher richness in the midstream and upstream of the Merbok Estuary (Zones B and C), as revealed by the Chao1 and Shannon indices. The sample-based and read number-based MOTU accumulation curves displayed direct relationship of diversity with number of samples and read numbers, respectively (Fig. 6; Supplementary Fig. 2), with higher detections observed in the 12S metabarcoding assay. The sample-based MOTU accumulation curves for both assays did not reach a plateau, suggesting that additional samples are needed to fully characterise the fish community in the study area. NMDS ordination plots for both metabarcoding assays, based on zones and pooled samples, exhibit partial to total overlap among fish communities (Fig. 7). Comparing MOTUs on a pair-wise basis revealed considerable disparities in MOTU diversity among the three zonations (PERMANOVA, Bray–Curtis, *p* < 0.001) (Table 1). In the ordination of the COI metabarcoding data, Zone A and Zone C community clusters were separated, with the inertia ellipse of Zone B partially overlapping both clusters. Within the 12S assay community analysis, Zone A, on the other hand, has a substantially broader community ellipse that encompasses both Zone B and Zone C clusters. The PERMANOVA analysis returned significantly different variances among the three zones (Zones A, B, and C) but minimal differentiation based on salinity measurement at all sampling sites (Supplementary Table 1).

**Table 1:** PERMANOVA results for the zones division and site salinity levels in fish community composition. Analysis was calculated using Bray–Curtis distances for both COI and 12S metabarcoding assays.

	*df*	Sum of Sqs	Mean Sqs	*F*	*R*^*2*^	*p*-value
**COI**						
Zone	2	1.82060	0.91032	8.70450	0.25061	0.001***
Salinity	1	0.21530	0.21526	2.05830	0.02963	0.053
Residual	50	5.22900	0.10458		0.71976	
Total	53	7.26490			1.00000	
**12S**						
Zone	2	0.57180	0.28590	6.52800	0.20158	0.001***
Salinity	1	0.07502	0.07502	1.71300	0.02645	0.063
Residual	50	2.18980	0.04380		0.77197	
Total	53	2.83662			1.00000	

***p < 0.001.

## Discussion

We investigated the efficiency of the eDNA metabarcoding approach in estimating fish diversity of a tropical brackish environment and compared it to previous conventional survey approaches. The feasibility and potential of eDNA metabarcoding as a rapid tool in assessing fish diversity in Merbok Estuary were demonstrated. Metabarcoding overcomes some negative aspects of conventional survey methods (e.g., the use of invasive/lethal sampling gears that often disturbs communities), resulting in negligeable impact on the natural ecosystem^41^. Furthermore, the various types of conventional sampling gears have considerable disparities in sampling effectiveness when compared to multi-locus eDNA metabarcoding assays^42,43^. In this regard, eDNA-based metabarcoding methods provide a non-invasive and cost-effective alternative for surveying aquatic ecosystems, and species with conservation interest. But its efficiency is contingent on the availability of a comprehensive and reliable genetic reference database for species identification. Furthermore, we also obtained in this study some unexpected findings such as the detection of species unlikely to be present in the surveyed areas and the non-detection of previously recorded species (albeit a moderate proportion). We discuss all these points below.

### Fish diversity detected via conventional surveys versus eDNA metabarcoding

Our results lend further support to the use of an eDNA metabarcoding approach on water samples collected from the Merbok Estuary at capturing the diversity of its fish community over time-consuming conventional surveys; a finding noted in other similar studies^29,31,44^. As illustrated, the eDNA metabarcoding assays detected ~ 82% of the fish families previously recorded during the last decade in Merbok Estuary by traditional surveys and detected additional 111 species (i.e., residents, migrants, or frequenters) inhabiting Merbok Estuary, within just a two-day sampling period. Several were new records. Some of these species are regarded to be in larval stage and thus likely uncaptured using the sampling gears employed in previous surveys. Furthermore, taxonomic identification of larva is very challenging even when sampled^17^. With the capacity to detect and identify species at any stage of development (from eggs to adults), the eDNA metabarcoding approach improves the accuracy of community composition estimation and provides additional information on community structure^44^. Thus, the eDNA assays provide a quick and more complete estimation of an aquatic ecosystem diversity through a powerful detection efficacy^28^.

The species detected by eDNA metabarcoding reflect the estuarine-mangrove community in Merbok Estuary, including estuarine residents (e.g., *Butis butis*, *Lates calcarifer*, *Epinephelus coioides*), marine-estuarine-dependent species that include amphidromous species (e.g., *Batrachomoeus trispinosus*, *Acentrogobius caninus*, *Siganus guttaus*), marine species and marine migrants that use the estuary as a nursery and/or feeding grounds (e.g., *Lutjanus johnii*, *Caranx ignobilis*, *Eleutheroma tetradactylum*), and estuarine-freshwater-dependent species (e.g., *Scatophagus argus*)^7,12,45^.

### Limitation one: absence of a comprehensive genetic reference database

Despite its potential to uncover higher diversity in this estuary, we highlight here three limitations of the eDNA method from water samples. Firstly, the major challenge is the absence of a comprehensive reference fish database of the two genes in this area and in general, of tropical fish communities. This is particularly true for megadiverse taxonomic tropical hotspot regions such as Merbok, where cryptic diversity is high^7^. Based on historical literature review of formal and informal documentations (e.g.,^46–49^), more than 500 species of fish could occur in Merbok region (coastal and brackish) with many of them being small and cryptic and not yet genetically examined. Furthermore, the significant number of misidentified species sequence entries in GenBank^50^ results in possible erroneous taxonomic assignment in the eDNA samples^51^. Reference sequence database, therefore, need to be expanded with curated sequences for increasing the amount of accurate metabarcoding-based identifications.

In recent years, ecosystem managers are turning to eDNA metabarcoding to glean information about community composition and diversity. The target loci (i.e., COI and 12S) were purposely selected because they are frequently employed in DNA barcoding and phylogenetic studies^7,52–54^. However, there remain significant gaps in the reference database of several taxon groups which hampers the optimal utility of eDNA metabarcoding^52^. Thus, future inventory tasks must prioritize the generation of reference sequences to include more annotated sequences in global databases (e.g., BOLD^55^ and NCBI GenBank^48^). The establishment of high-quality reference databases of local biodiversity should be a prerequisite in DNA-based biodiversity monitoring. We have established a COI-based barcode of 134 fish species occurring in Merbok Estuary in a previous study^7^. Approximately 37% of the species (21 out of 57 species) found in the COI metabarcoding assay matched the regional reference database established in^7^ (Supplementary Table 2). At least 36 species identified in the COI metabarcoding assay are not yet included in the local reference database. This moderate agreement with the database in^7^ indicates that barcoding efforts in the Merbok Estuary still need to be expanded. Barcoding studies on the region's ichthyodiversity are underway, including sequences generated from other mitochondrial markers (e.g., 12S and cytochrome *b*). This will elevate the efficacy of eDNA metabarcoding and offers the opportunity to re-analyse sequences generated in this study against updated databases. The eDNA dataset generated in this study forms a baseline for future studies and it will be valuable for estimating the Merbok fish community composition changes across time.

### Limitation two: detection of metabarcodes of species unexpected to occur in the studied area, likely due to drifting of tissue materials

The amount of eDNA released into the environment varies by species and life stage and its degradation is related to abiotic (e.g., salinity, temperature, hydrological conditions, UV radiation and pH) and biotic (e.g., microbial activity and extracellular enzymes) environmental variables^56^. Water movements from adjacent freshwater and marine bodies to the Merbok Estuary, could potentially transport eDNA of non-occurring species into the estuary^36,42^. This possibility needs to be considered when assessing the diversity of fish communities along a heterogeneous spatial gradient, such as estuary and mangrove ecosystems^57^. Although our PERMANOVA analysis revealed significant variance among the three zones (Zones A, B, and C), few seemingly non-resident species were detected.

Metabarcodes of species from the strictly freshwater families Cyprinidae, Anabantidae, Channidae, Osphronemidae, Aplocheilidae and Poeciliidae were detected within the estuary. None of these have been recorded in previous surveys within the estuary but are known to occur upstream, in the freshwater part of the Merbok River and Muda River basins. For instance^58^, recorded the presence of the blue panchax, *Aplocheilus armatus* (Aplocheilidae), and the dwarf snakehead, *Channa limbata* (Channidae), in the freshwater streams of Gunung Jerai Forest Reserve, located ~15 km north of Merbok Estuary. We opine that the presence of metabarcodes of these two species is certainly due to the downstream water movement into the estuary and does not reflect the presence of these species there. A recent diversity survey conducted in the streams of Ulu Muda Forest Reserve (located within the Muda basin and connected to the Merbok Estuary), located ~80 km northeast of our study area, recorded several species that correspond to our eDNA metabarcodes such as *Barbodes binotatus*, *Mystacoleucus obtusirostris*, *Tor tambra*, *Anabas testudineus*, and *Channa striata*^59^. Similarly, the detection of coral reef endemic species is likely due to eDNA transportation into the estuary by oceanic and tidal currents. This is supported by the timing of our sampling during high tide, when the ocean level reaches its maximum and seawater massively enters the estuary. ^22^ demonstrated that species from the surrounding marine and freshwater habitats are more easily detected during this period. Given the large marine and freshwater ichthyodiversity in this region with more than 1400 species recorded, the detection of only few species that are supposed not occurring in the Merbok Estuary is a marginal concern.

The detection of threatened and endangered species is another prominent objective for eDNA applications because less efficient traditional approaches using harmful fishing gears are not adapted to monitor their presence^60^. Previous studies highlighted the advantages of eDNA approaches for detecting rare and/or valuable species^61,62^. Our metabarcoding assays found a few species of conservation importance including the endangered blacktail reef shark *Carcharhinus amblyrhynchos* and the near threatened blacktip reef shark *Carcharhinus melanopterus*, none of which had been previously recorded in the study area. These two species prefer to live nearby coral reefs. The blacktail reef shark was one of the common catches in Peninsular Malaysia a few decades ago based on a report on Malaysian shark fisheries^63^, before its catches considerably declined. Apparently, this species still occurs in the Merbok region. The surprising discovery of these threatened species illustrates the value of eDNA metabarcoding as a tool for assessing their conservation status and for monitoring the biodiversity of fragile ecosystems like the estuaries and mangroves^23,41^.

### Limitation three: eDNA metabarcoding methods may not successfully capture whole species diversity

Despite its enormous potential, some taxa still go undetected due to several inherent limitations of the metabarcoding assay^64^. The current study missed nine families previously identified by conventional surveys. This may be attributed to the following reasons: low density of individuals in the environment leading to non-detectable amount of eDNA in the water sample^65,66^, rapid eDNA degradation^67^, primer specificity^36^, or missing information on the reference database. Considering the last reason, although COI reference sequences are available in GenBank, albeit not in large numbers, our assessment has shown that there are still significant gaps in the 12S sequence database for fishes. At least four species of the families recorded in the conventional surveys are not present in the 12S reference database (i.e. *Chiloscyllium indicum*, *Dussumieria albulina, Neostethus lankesteri,* and *Pseudorhombus elevatus*). These gaps need to be addressed to improve the resolution and accuracy of species assignment, specifically with the generation of more 12S sequences for endemic and commercial fish species.

Often eDNA metabarcoding methods exhibit some degree of taxonomic selectivity due to primer specificity. Different aquatic ecosystems and fish communities require the use of different primers to obtain reliable eDNA metabarcoding data. Before implementing eDNA metabarcoding on a broad scale for fish monitoring, it is necessary to evaluate the performance of different primer pairs (in silico and in vitro) in relation to the local biodiversity^64^. The efficiency of the metabarcoding approach is highly dependent on the selection of primers used in the initial amplification. Numerous studies have examined the efficacy of universal primers for characterising fish diversity, either in silico, in experimental tanks, or in the wild. For example^64^, evaluated the performance of 22 primer sets (i.e., two COI, seven 12S, six 16S, and seven cytochrome *b* primer sets) in the taxonomic classification of a rich fish community in 104 water bodies in Beijing via eDNA metabarcoding. They discovered that primers targeting the 12S gene generally detect greater fish diversity compared to other markers.

The ideal metabarcoding marker would have a comprehensive reference library. Such a database is available for the COI marker, but because of the low specificity of its primer pair, it requires other complementary markers; the 12S marker, on the other hand, has excellent primer specificity but insufficient sequence references. Therefore to reduce the combined impact of primer selectivity and library quality, it is recommended to use different DNA markers having each a primer set to improve taxonomic detection and identification^66–69^. Two genetic markers were used in our study to overcome the drawbacks of using a single primer set in the metabarcoding assays. We utilized COI and 12S markers in our eDNA assays, in reference to previous metabarcoding studies^29,70,71.^

Our study showed that the COI assay produced higher number of reads with broader detections across taxa when compared to the 12S assay, which is similar to the findings of^29,70^. However, 96.1% of the total COI reads were assigned to non-fish taxa. Mitochondrial COI is often the marker of choice for many barcoding and metabarcoding applications due to its large reference library, but the performance of COI in targeting fish taxa in environmental samples is not always optimal, as shown by our results. A recent study in the United Kingdom also evaluated the specificity and reproducibility of four primer pairs (three COI and one 12S pairs) in assessing marine and freshwater fishes using eDNA metabarcoding^72^. Due to the non-specific amplification of non-targeted prokaryotic and eukaryotic DNA, the COI primers performed significantly less favourably than the 12S primers in characterising fish diversity^72^. Despite being the standard fish barcoding gene^73^, the COI gene does not appear to be the ideal choice as a metabarcoding marker because of the low specificity of its available primers. Notwithstanding the wider taxonomic coverage of COI sequences compared to other loci, the high variability of the COI sequences complicates the design of universal fish-specific primers for short amplicons, limiting the capacity of COI for eDNA biodiversity assessment^72^. In comparison to the COI assay, 12S assay retrieved a higher number of fish species. The 12S MiFish primers^74^ are reputed to be teleost specific and have been successfully employed in other eDNA surveys over a wide range of aquatic environments, including estuaries^22,24^, marine^44,75^, and freshwater ecosystems^64,76^.

### Diversity patterns and composition

Biodiversity is inextricably linked to environment, especially in estuaries and mangrove ecosystems^77^. Based on the ecological characteristics of ecosystem landscapes in Merbok Estuary, three zones were pre-defined (i.e., Zone A, Zone B, and Zone C). These three zones have also different degrees of anthropogenic impact with the lowest disturbances in Zone B. Zone B harbours the highest diversity estimates based on the Chao1 and Shannon’s indices using the 12S assay (Fig. 5b). Our findings agree with^16^ who likewise found the greatest abundance of fish species in the midstream of the Merbok Estuary. This is an indicator that high biodiversity warrants a pristine environment^22^. The NMDS ordination allied with PERMANOVA based on read counts of the two metabarcoding assays, confirmed the clustering results indicating that each of the three designated zones supports a distinct fish community. The results of the PERMANOVA showed that salinity varied only minimally across sampling sites in the Merbok Estuary (Fig. 1, Supplementary Table 1) and had no influence on the structuring of the fish community.

Fish communities respond to many environmental variables including salinity, water temperature, food availability, and substrate type to determine their distribution throughout the aquatic habitats, especially in estuaries and mangrove ecosystems^78^. Apart from the variations observed in the zones, no other notable spatial trends in the MOTUs read abundance across the Merbok Estuary were observed. However, a detailed spatial and geographical comparison of fish communities requires a more extensive sampling coverage, a higher sample density, and comprehensive physicochemical data collection^22,26^, which was out of the scope of our study. Even so, a recent study demonstrated that species assemblage composition differed noticeably across different environments on a small geographic scale, indicating the specificity of eDNA signals despite significant water mixing^26^. Even though our study has shown that eDNA provides valuable information on the community composition of different habitats within a specific region, complementary conventional approaches with better spatial fidelity are needed to confirm our species-distribution results along the Merbok estuary. Furthermore, conventional surveys have a distinct advantage when eDNA assays cannot resolve sequence diversity down to the species level and when biological data (e.g., number of individuals, size, weight, fecundity, and other critical aspects of life history) need to be collected. Thus, we advocate incorporating eDNA metabarcoding sampling into traditional surveys as a best practice for performing whole biodiversity surveys in a complex ecosystem like the estuaries and the mangroves^54^ demonstrated that combining both eDNA and conventional approaches uncovers a broader taxa diversity and provides a more holistic perspective on species compositions in an aquatic environment.

## Conclusion

This is one of the first studies aiming to evaluate the practicability and value of an eDNA metabarcoding approach to assess fish diversity in a tropical mangrove estuarine environment in Southeast Asia. Our findings demonstrated strong advantages of using such approach, albeit with some limitations. We showed that eDNA metabarcoding is capable of rapidly capturing a large proportion of the fish diversity of a complex ecosystem within a short sampling period, including taxa of conservation importance. However, the absence of a comprehensive and reliable genetic database for comparison, the transportation of exogen eDNA by water movements from surrounding areas, and the selectivity of primers need to be recognised as limitations and addressed. Overall, this study confirms that the fish diversity of Merbok Estuary is extremely rich and more studies are needed to fully document it.

## Methods

### Ethics statement

The study was conducted following the relevant national and international guidelines. Since the experiments were performed only with water samples and did not involve any endangered/ protected fish species or experiments on live specimens, an ethics statement is not required for this study. This study was carried out following the recommendations and approval by the Universiti Sains Malaysia Animal Ethics Committee. All methods were performed in accordance with the relevant guidelines and regulations.

### Merbok Estuary capture records

A compiled checklist of fish species in Merbok Estuary was constructed from our previous and other recent surveys^7,12,16^. We also included their conservation status as according to the International Union for Conservation of Nature (IUCN) Red List (https://www.iucnredlist.org/), habitat, pelagic zone inhabited, and migration type (where available).

### Study sites and water sample collections

Field samplings were conducted at Merbok Estuary, Kedah, northwest Peninsular Malaysia (Fig. 1). The Merbok Estuary spans approximately 30 km with a catchment area of about 550 km2 and supports approximately 40 km^2^ of mangrove forest^12^. The sampling activity was conducted on 21 and 22 February 2018, coinciding with the dry season in this region with estimated monthly rainfalls between 110 and 170 mm^79^. The sampling sites along the estuary were categorised into three zones: Zone A (Merbok Estuary; KM 1.0–KM 10.0 from the open ocean); Zone B (Midstream Merbok River (KM 10.0–KM 20.0); and Zone C (KM 20.0–KM 30.0). Zone A covers the area of the estuary and coastal beaches. Two sand beaches – Pantai Tanjung Dawai and Pantai Merdeka are located within this area. These areas are impacted by tourism activities, commercial fisheries, and local housing. Located in the midstream, the vegetation in Zone B is the least impacted with the lowest anthropogenic disturbance (although water quality in Zone B may be impacted from upstream), with most intertidal zones covered by pristine mangroves. Zone C is the most disturbed area with probable pollution, evident from the observation of floating debris and oily substances from nearby residential and agricultural areas. One of the main tourist attraction sites, the Semeling Jetty Tourist Complex, is located in this area.

A total of 54 sampling sites, 18 from each zone, were surveyed (Supplementary Table 1). Water samples were collected during high tide from three positions along a horizontal transect of the river: one from each bank of the estuary, and the third from the middle of the estuary. Such design is postulated to maximise the spatial coverage of the collection area. Using sterile disposable plastic bottles, one litre of water was collected at a depth of 1.0 m at each site. Sampling was carried out from the mouth—point A1 to the upstream—point C6. Water samples were collected from the bow of the boat to minimise the risk of contamination from the boat itself. Three negative field controls were included, one for each zone. All sampling equipment, including the sample bottles and the water sampler, were cleaned using a 10% solution of commercial bleach (~ 7.4% sodium hypochlorite), rinsed with distilled water, and wiped clean with 70% ethanol to minimise cross-site and exogenous DNA contamination. Disposable gloves were replaced between all sampling sites to reduce the risk of cross-contamination between samples. Water temperature (data not shown) and salinity (Fig. 1, Supplementary Table 1) were measured with a multiparameter instrument (YSI Pro 1030). The probe of the multiparameter instrument was cleaned with distilled water and wiped with a lint-free cleaning cloth before each measurement. Measurements were carefully taken at the stern of the boat to avoid possible cross-contamination with collected water samples. All water samples were packed in individual plastic bags and immediately stored on ice for transport.

### Laboratory contamination control

Filtration, DNA extraction, pre-PCR, and post-PCR procedures were conducted in four separate facilities, where no other environmental or organismal samples were processed. Working surfaces were decontaminated before use with a 10% bleach solution. Only sterilised consumables (e.g., filter capsules, syringes, gloves, tubes, and tips) were used throughout the experiment, and pipettes were decontaminated using 10% bleach solution and 70% ethanol before every use.

### Filtration and DNA extraction

All water samples were filtered using Sterivex™-GV Sterile Vented Filter Unit, 0.22 μm polyethersulfone membrane, within 12 hours of sampling. For each of the three designated zones, one negative control (1 L of pure water) was included and filtered to monitor contamination during the filtration, extraction, and subsequent DNA library preparations. After filtration, all membranes were individually stored in a sterile sample bag with silica beads and preserved at − 20 °C until extraction. DNA was extracted from the filter membrane using a DNeasy Blood and Tissue Kit (Qiagen) with slight modifications from the manufacturer’s protocol: 540 µL of ATL lysis buffer and 60 µL of Proteinase K were added to each sample, and the incubation at 56 °C was extended to 3 hours. The final elution was done in 80.0 µL volume. Then, all three filtered negative controls were extracted in the same way as the field samples. The extracted DNA was then transferred into a new labelled tube, secured with parafilm, and stored at – 20 °C until further use. The DNA extract concentration was quantified using Qubit dsDNA HS Assay (Invitrogen, USA).

### Library preparation and amplicon sequencing

PCR metabarcoding assays were employed using two mitochondrial genetic markers, the cytochrome oxidase I (COI) and 12S rRNA (12S) genes. The COI gene was amplified using the metazoan universal primers mICOIintF^80^ and jgHCO2198^81^, targetting a 313 base pairs (bp) fragment of the variable region (Table 2). Meanwhile, the 12S gene was amplified using the primer pair MiFish-U^74^, targeting teleost fish which rendered amplicons of approximately 170 bp in size (Table 2). A two-step PCR was conducted to prepare the libraries for Illumina MiSeq sequencing. Contamination was monitored using negative controls for all experiments.

**Table 2:** Illumina adapters and primers used in the metabarcoding assay.

	Oligonucleotide sequence (5′–3′)	References
**Adapters**		
Forward overhang	TCGTCGGCAGCGTCAGATGTGTATAAGAGACAG- [locus-specific sequence]	^82^
Reverse overhang	GTCTCGTGGGCTCGGAGATGTGTATAAGAGACAG- [locus-specific sequence]	
**Primers**		
COI		
mlCOIintF	GGWACWGGWTGAACWGTWTAYCCYCC	^80^
jgHCO2198	TAIACYTCIGGRTGICCRAARAAYCA	^81^
12S		
MiFish-U-F	GTCGGTAAAACTCGTGCCAGC	^74^
MiFish-U-R	CATAGTGGGGTATCTAATCCCAGTTTG	

For preparing the COI and 12S libraries, we adapted the “16S Metagenomic Sequencing Library Preparation Protocol”^82^. The first PCR was performed to amplify both regions with an overhanging linker sequence (Table 2) for each Nextera XT index (Illumina, USA). PCR amplifications were done in triplicates for each sample. The PCR mixture for COI (20 μL) contained 10.0 μL of KAPA HiFi HotStart ReadyMix (2X), 1.0 μL of each primer (5.0 μM), 0.3 μL of BSA (20 µg/µL), 5.7 μL nuclease-free water, and 2.0 μL of DNA template. The PCR reaction started with denaturation at 95 °C for 10 min followed by 35 cycles at 94 °C for 1 min, 46 °C for 1 min, and 72 °C for 1 min, and a final extension at 72 °C for 5 min. Meanwhile, the 12S PCR mixture (20 μL) contained 10.0 μL of KAPA HiFi HotStart ReadyMix (2X), 1.2 μL of each primer (5.0 μM), 4.1 μL nuclease-free water, and 3.5 μL of DNA template. The PCR cycle profile was set with an initial 3 min denaturation at 95 °C, followed by 35 cycles of 20 s denaturation at 98 °C, 25 s annealing at 66 °C, and 17 s extension at 72 °C, and a final 5 min extension at 72 °C. Successful PCR replicates were pooled and purified using the AMPure XP kit (Beckman Coulter, USA).

All samples were quantified using Qubit dsDNA HS Assay (Invitrogen, USA). In addition to the 54 individual samples, equimolar amounts of PCR products from each sample, derived from each site, were pooled before the second index PCR for a total of 59 libraries per assay. Pooling strategy of the additional five samples is described in Table 3. These five additional samples were included to increase the MOTU discovery from the collected individual samples. All samples were then amplified in the second index PCR following the Nextera XT index kit (Illumina, USA) protocol. All samples were also purified using the AMPure XP kit (Beckman Coulter, USA) and validated using Agilent TapeStation 4200. The library concentration and quality were measured by staining procedure (Quanti-iT™ Pico Green dsDNA Assay, Invitrogen USA) and spectrophotometry (Nanodrop, Thermo Scientific, USA). Both COI and 12S amplicon libraries were separately sequenced on an Illumina MiSeq platform (Illumina, Singapore) with COI amplicons running at 2 x 300 bp paired-end format and the 12S amplicons at 2 × 150 bp paired-end format, following the manufacturer’s instructions.

**Table 3:** Descriptions of pooling mechanism for each sample pool before the second index PCR.

Sample #	Sample pool	Sample descriptions
1	Pool Zone A (TA)	3.0 µL was taken from each homogenised sample (first PCR product) from Zone A (18 samples from Zone A × 3.0 µL = 54.0 µL)
2	Pool Zone B (TB)	3.0 µL was taken from each homogenised sample (first PCR product) from Zone B (18 samples from Zone B × 3.0 µL = 54.0 µL)
3	Pool Zone C (TC)	3.0 µL was taken from each homogenised sample (first PCR product) from Zone C (18 samples from Zone C × 3.0 µL = 54.0 µL)
4	Total pool replicate 1 (T1)	1.0 µL was taken from each homogenised sample (first PCR product) from all zones – Zone A, B and C (54 samples × 1.0 µL = 54.0 µL)
5	Total pool replicate 2 (T2)	1.0 µL was taken from each homogenised sample (first PCR product) from all zones – Zone A, B and C (54 samples × 1.0 µL = 54.0 µL)

### Bioinformatic and data analyses

MiSeq read quality for all samples was assessed using FastQC^83^ with summary statistics for each run visualised in MultiQC^84^. Individual sequencing reads were demultiplexed and trimmed with quality filtering using BBDuk within the BBTools package (https://sourceforge.net/projects/bbmap). Sequences were further processed in USEARCH v11.0.667 (https://www.drive5.com/usearch/). Paired-end reads were aligned and merged. Pre-processed reads were then dereplicated, and all low abundance sequence clusters (< 1% of the total number of unique sequences) were removed from the subsequent analyses. Finally, the dereplicated sequences were clustered into molecular operational taxonomic units (MOTUs) at 97% threshold using UPARSE^85^ as implemented in USEARCH. Within the same analysis tool, chimeric reads were screened and removed. MOTUs represented by a single or double sequence(s) (i.e., singletons and doubletons) were discarded. BLASTn was used in querying the MOTUs against the NCBI GenBank database^48^. Each MOTU was assigned to a unique species based on sequence similarity greater than 97%, a cut-off threshold that minimises erroneous taxonomic assignments^74^.

The BLAST top hits (those with the highest identity with query sequence) were applied to each representative MOTU for species assignment. Stringent sequence and taxon filtering were employed to generate a dataset with high certainty. In order to reduce the possibility of introducing false-positive results, MOTUs represented by less than 0.02% of the generated reads were identified and discarded because they could be the consequence of contamination^36,86,87^. After removing low-frequency noise, spurious species assignments were inspected against the negative control annotation. The MOTUs corresponding to taxa detected in the negative controls were removed from the experimental samples. All the remaining MOTUs assigned to species were then manually evaluated. All MOTUs with detection hits of taxa outside of our targeted taxonomic groups of bony fish and elasmobranchs were omitted. Species identities were then cross-checked to the species distribution reported by FishBase (https://www.fishbase.se/), IUCN (https://www.iucnredlist.org/), Eschmeyer’s Catalog of Fishes^88^, and Sharks of the World Illustrated Guide^89^. Marine or freshwater species that are unlikely to inhabit the study areas or not previously recorded in the region (taxon exotic to the study area) were identified as false positive^36^ and were excluded in subsequent analyses. Bioinformatic analyses including BBTools, USEARCH, UPARSE, and QIIME were run using Unix shell script in a high-performance computing (HPC) workstation.

### Statistical analyses

Statistical analyses were performed in RStudio version 1.4.1106^90^ using several packages. Alpha (α) diversity analyses were carried out using the phyloseq package^91^. The MOTU bar plots and heatmaps based on the relative abundances (read counts) were computed with normalised datasets for both assays (COI = 2730; 12S = 113,809). MOTU richness (α-diversity) patterns among the three defined zones (Zones A, B, and C) and the pooled samples were based on non-parametric Chao1 and Shannon indices. Sample-based and read numbers-based MOTU accumulation curves was plotted with the specaccum function implemented in the vegan package^92^ to compare species richness detected from both assays. Beta (β) diversity was calculated using Bray-Curtis metric (function vegdist) within the vegan package. In each sample, read numbers of MOTUs were transformed into fourth square root values and translated into relative abundances for the Bray-Curtis dissimilarity calculations. Biodiversity patterns across the datasets were estimated with the non-metric multidimensional (NMDS) ordination using the vegan function metaMDS. Permutational multivariate analysis of variance (PERMANOVA) was calculated with 999 free permutations with function adonis in vegan to assess community composition divergence with zones and salinity level defined as factors. Plots from all performed analyses were visualised using ggplot2 package^93^.

## Supplementary Information

Supplementary Information.

## Data Availability

The data analysed in this study are available from the corresponding author upon reasonable request.
