# Supplementary material for: Assessing a megadiverse but poorly known community of fishes in a tropical mangrove estuary through environmental DNA (eDNA) metabarcoding
Source: Sci Rep. 2022 Sep 29;12:16346. doi: 10.1038/s41598-022-19954-3 (PMC9523059; doi:10.1038/s41598-022-19954-3)
Supplement: Supplementary file 1 — Supplementary Information. [file 41598_2022_19954_MOESM1_ESM.docx]

**Supplementary information**


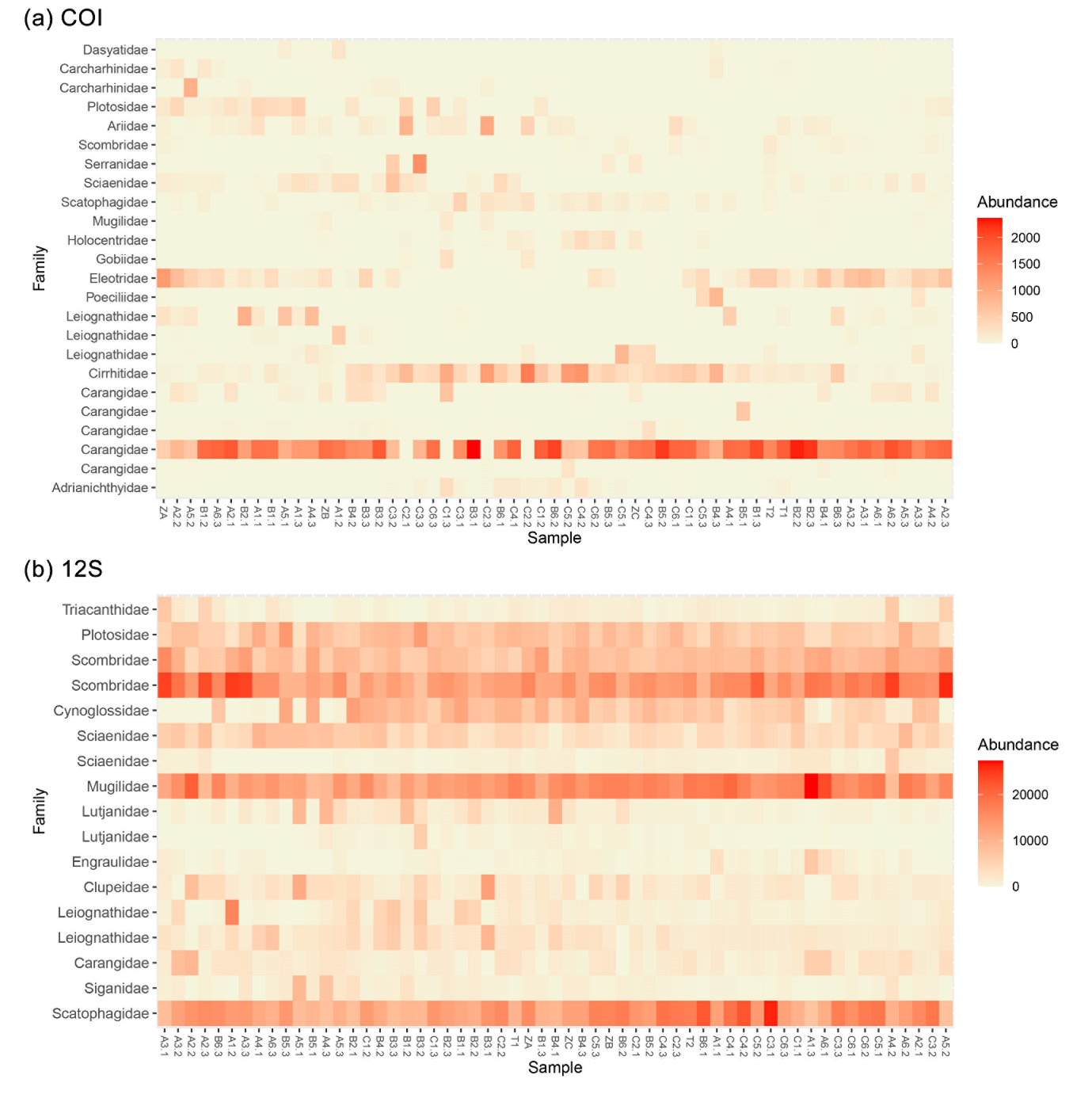


**Supplementary Figure 1.** Heatmaps of the read abundance within each sample featuring the top 50% families detected from both metabarcoding assays: (a) COI and (b) 12S.

**
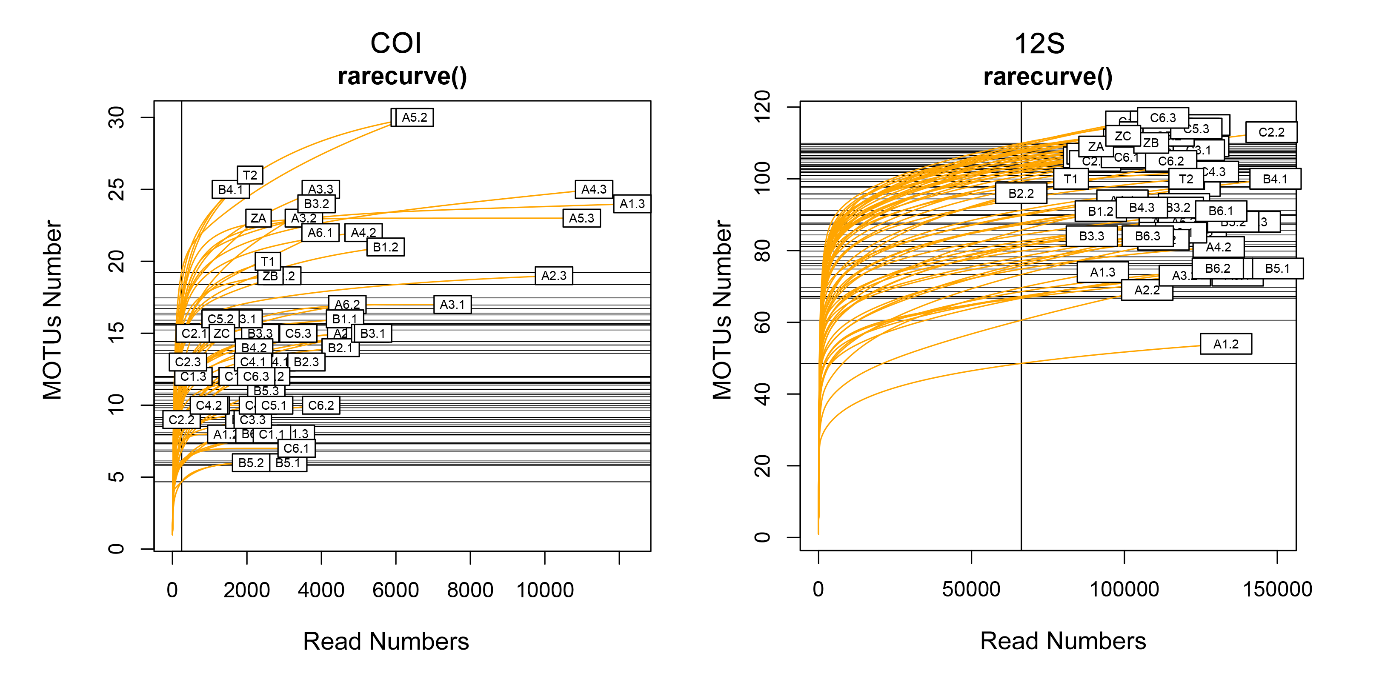
**

**Supplementary Figure 2.** MOTU accumulation curves based on read numbers representing the number of fish MOTUs identified in all samples analysed by eDNA metabarcoding assays: (a) COI assay and (b) 12S assay.

**Supplementary Table 1.** List of sampling sites within the sampling transects at the three designated zones with collection metadata.

| **Zone** | **Sampling transects** | **Sampling sites** | **GPS coordinates** | **Salinity (ppt)** | **Collection date** |
| --- | --- | --- | --- | --- | --- |
| **A** | **A1** | **A1.1** | 05° 40’ 26.0’’N, 100° 20’ 37.8’’E | 30.0 | 21/02/2018 |
|  |  | **A1.2** | 05° 40’ 44.2’’N, 100° 20’ 31.7’’E | 29.0 | 21/02/2018 |
|  |  | **A1.3** | 05° 41’ 12.1’’N, 100° 20’ 45.2’’E | 34.0 | 21/02/2018 |
|  | **A2** | **A2.1** | 05° 40’ 55.9’’N, 100° 21’ 29.0’’E | 36.0 | 21/02/2018 |
|  |  | **A2.2** | 05° 40’ 27.3’’N, 100° 21’ 28.4’’E | 35.0 | 21/02/2018 |
|  |  | **A2.3** | 05° 40’ 05.3’’N, 100° 21’ 15.7’’E | 29.0 | 21/02/2018 |
|  | **A3** | **A3.1** | 05° 40’ 09.2’’N, 100° 22’ 00.9’’E | 33.0 | 21/02/2018 |
|  |  | **A3.2** | 05° 40’ 26.9’’N, 100° 22’ 17.6’’E | 34.5 | 21/02/2018 |
|  |  | **A3.3** | 05° 40’ 41.5’’N, 100° 22’ 27.6’’E | 34.0 | 21/02/2018 |
|  | **A4** | **A4.1** | 05° 39’ 59.1’’N, 100° 23’ 01.7’’E | 33.0 | 21/02/2018 |
|  |  | **A4.2** | 05° 39’ 52.4’’N, 100° 22’ 54.3’’E | 36.0 | 21/02/2018 |
|  |  | **A4.3** | 05° 39’ 40.3’’N, 100° 22’ 43.5’’E | 36.0 | 21/02/2018 |
|  | **A5** | **A5.1** | 05° 39’ 25.1’’N, 100° 23’ 03.0’’E | 36.0 | 21/02/2018 |
|  |  | **A5.2** | 05° 39’ 37.6’’N, 100° 23’ 14.0’’E | 36.0 | 21/02/2018 |
|  |  | **A5.3** | 05° 39’ 51.6’’N, 100° 23’ 17.0’’E | 36.0 | 21/02/2018 |
|  | **A6** | **A6.1** | 05° 39’ 38.1’’N, 100° 23’ 48.4’’E | 36.0 | 21/02/2018 |
|  |  | **A6.2** | 05° 39’ 28.7’’N, 100° 23’ 51.2’’E | 37.0 | 21/02/2018 |
|  |  | **A6.3** | 05° 39’ 19.6’’N, 100° 23’ 50.5’’E | 34.0 | 21/02/2018 |
| **B** | **B1** | **B1.1** | 05° 38’ 35.6’’N, 100° 23’ 59.8’’E | 28.5 | 21/02/2018 |
|  |  | **B1.2** | 05° 38’ 35.4’’N, 100° 24’ 07.7’’E | 35.0 | 21/02/2018 |
|  |  | **B1.3** | 05° 38’ 38.0’’N, 100° 24’ 23.6’’E | 30.0 | 21/02/2018 |
|  | **B2** | **B2.1** | 05° 38’ 10.0’’N, 100° 24’ 20.7’’E | 36.0 | 21/02/2018 |
|  |  | **B2.2** | 05° 38’ 01.9’’N, 100° 24’ 17.8’’E | 36.0 | 21/02/2018 |
|  |  | **B2.3** | 05° 37’ 54.2’’N, 100° 24’ 21.0’’E | 37.0 | 21/02/2018 |
|  | **B3** | **B3.1** | 05° 38’ 11.3’’N, 100° 24’ 56.5’’E | 37.0 | 21/02/2018 |
|  |  | **B3.2** | 05° 38’ 19.0’’N, 100° 24’ 54.6’’E | 36.0 | 21/02/2018 |
|  |  | **B3.3** | 05° 38’ 29.4’’N, 100° 24’ 51.9’’E | 36.0 | 21/02/2018 |
|  | **B4** | **B4.1** | 05° 38’ 35.9’’N, 100° 25’ 34.2’’E | 35.0 | 21/02/2018 |
|  |  | **B4.2** | 05° 38’ 27.9’’N, 100° 25’ 35.1’’E | 35.0 | 21/02/2018 |
|  |  | **B4.3** | 05° 38’ 21.1’’N, 100° 25’ 33.0’’E | 34.0 | 21/02/2018 |
|  | **B5** | **B5.1** | 05° 38’ 01.6’’N, 100° 26’ 00.7’’E | 31.0 | 22/02/2018 |
|  |  | **B5.2** | 05° 38’ 06.4’’N, 100° 26’ 04.2’’E | 30.0 | 22/02/2018 |
|  |  | **B5.3** | 05° 38’ 14.2’’N, 100° 26’ 02.9’’E | 29.0 | 22/02/2018 |
|  | **B6** | **B6.1** | 05° 38’ 42.9’’N, 100° 26’ 30.1’’E | 31.0 | 22/02/2018 |
|  |  | **B6.2** | 05° 38’ 37.2’’N, 100° 26’ 33.2’’E | 32.0 | 22/02/2018 |
|  |  | **B6.3** | 05° 38’ 36.5’’N, 100° 26’ 39.9’’E | 32.0 | 22/02/2018 |
| **C** | **C1** | **C1.1** | 05° 39’ 28.5’’N, 100° 27’ 00.2’’E | 32.0 | 22/02/2018 |
|  |  | **C1.2** | 05° 39’ 27.1’’N, 100° 26’ 53.9’’E | 31.5 | 22/02/2018 |
|  |  | **C1.3** | 05° 39’ 26.9’’N, 100° 26’ 49.2’’E | 32.0 | 22/02/2018 |
|  | **C2** | **C2.1** | 05° 40’ 12.5’’N, 100° 27’ 03.2’’E | 32.0 | 22/02/2018 |
|  |  | **C2.2** | 05° 40’ 08.0’’N, 100° 27’ 06.0’’E | 33.0 | 22/02/2018 |
|  |  | **C2.3** | 05° 40’ 03.1’’N, 100° 27’ 07.8’’E | 31.5 | 22/02/2018 |
|  | **C3** | **C3.1** | 05° 40’ 48.6’’N, 100° 27’ 26.8’’E | 31.0 | 22/02/2018 |
|  |  | **C3.2** | 05° 40’ 50.3’’N, 100° 27’ 23.3’’E | 31.0 | 22/02/2018 |
|  |  | **C3.3** | 05° 40’ 53.9’’N, 100° 27’ 21.4’’E | 30.0 | 22/02/2018 |
|  | **C4** | **C4.1** | 05° 41’ 01.8’’N, 100° 28’ 08.2’’E | 30.0 | 22/02/2018 |
|  |  | **C4.2** | 05° 40’ 59.3’’N, 100° 28’ 07.7’’E | 30.0 | 22/02/2018 |
|  |  | **C4.3** | 05° 40’ 57.5’’N, 100° 28’ 08.3’’E | 28.0 | 22/02/2018 |
|  | **C5** | **C5.1** | 05° 41’ 13.9’’N, 100° 28’ 50.6’’E | 30.0 | 22/02/2018 |
|  |  | **C5.2** | 05° 41’ 14.9’’N, 100° 28’ 51.1’’E | 30.0 | 22/02/2018 |
|  |  | **C5.3** | 05° 41’ 18.8’’N, 100° 28’ 51.8’’E | 30.0 | 22/02/2018 |
|  | **C6** | **C6.1** | 05° 41’ 20.0’’N, 100° 29’ 15.6’’E | 28.5 | 22/02/2018 |
|  |  | **C6.2** | 05° 41’ 20.6’’N, 100° 29’ 17.6’’E | 29.0 | 22/02/2018 |
|  |  | **C6.3** | 05° 41’ 21.3’’N, 100° 29’ 19.3’’E | 29.0 | 22/02/2018 |

**Supplementary Table 2.** Fish taxa (at the order, family and species levels) detected from eDNA by COI and 12S metabarcoding assays.

| **Order** | **Family** | **Species** | **Common name** | **IUCN status^a^** | **Habitat^b^** | **Pelagic zone^c^** | **Migration^d^** | **Notes** | **Merbok CR^e^** | **eDNA assay** |
| --- | --- | --- | --- | --- | --- | --- | --- | --- | --- | --- |
| Carcharhiniformes | Carcharhinidae | *Carcharhinus amblyrhynchos* | Blacktail reef shark | EN | M | R | O | Endangered |  | COI |
|  |  | *Carcharhinus melanopterus* | Blacktip reef shark | NT | B, M | R | A | Near threatened |  | COI |
| Myliobatiformes | Dasyatidae | **^Ƀ^***Brevitrygon walga* | Scaly whipray | NT | M | D |  | Near threatened | X | COI |
|  | Gymnuridae | **^Ƀ^***Gymnura poecilura* | Long-tailed butterfly ray | NT | M | D |  | Near threatened | X | COI |
| Acanthuriformes | Siganidae | *Siganus fuscescens* | Mottled spinefoot | LC | M | R | O |  | X | 12S |
|  |  | *Siganus guttatus* | Orange-spotted spinefoot | LC | M, B | R |  |  | X | 12S |
|  |  | *Siganus sutor* | Shoemaker spinefoot | LC | B, M | R |  |  |  | COI |
|  | Leiognathidae | *Photopectoralis bindus* | Shortnose ponyfish | NE | B, M | D | AM |  | X | 12S |
|  |  | *Leiognathus equula* | Common ponyfish | LC | F, B | D | AM |  | X | 12S |
|  |  | **^Ƀ^***Deveximentum ruconius* | Deep pugnose ponyfish | NE | F, B, M | D | AM |  | X | COI, 12S |
|  |  | ********Deveximentum* sp. | Ponyfish |  |  |  |  | BLAST detection as *Leiognathus* cf. *ruconius*. Refer to Note^1^. |  | COI |
|  |  | *Photopectoralis bindus* | Orangefin ponyfish | NE | B, M | D | A |  |  | COI |
|  | Lobotidae | *Lobotes surinamensis* | Tripletail | LC | B, M | B | O |  |  | COI |
|  | Scatophagidae | **^Ƀ^***Scatophagus argus* | Spotted scat | LC | F, B, M | R | AM |  | X | COI, 12S |
| Anabantiformes | Anabantidae | *Anabas testudineus* | Climbing perch | LC | F, B | D | P |  |  | 12S |
|  | Channidae | **Channa limbata* | Dwarf snakehead | LC | F | B |  | BLAST detection as *Channa gachua*. Refer to Note^2^. |  | 12S |
|  |  | *Channa striata* | Striped snakehead | LC | F, B | B |  |  |  | 12S |
|  | Osphronemidae | *Trichogaster lalius* | Dwarf gourami | LC | F | B |  |  |  | 12S |
|  |  | *Trichopodus trichopterus* | Three spot gourami | LC | F | B |  |  |  | 12S |
| Anguilliformes | Anguillidae | *Anguilla bicolor* | Indonesian shortfin eel | NT | F, B, M | D | C | Near threatened |  | 12S |
|  | Muraenidae | *Gymnothorax reticularis* | Moray eel | NE | M | D |  |  |  | 12S |
|  | Ophichthidae | *Neenchelys buitendijki* | Fintail serpent eel | NE | M | D |  |  |  | 12S |
|  |  | **Ophichthus lithinus* | Evermann’s snake eel | NE | M | B |  | BLAST detection as *Ophichthus evermanni*. Refer to Note^3^. |  | 12S |
| Aulopiformes | Synodontidae | *Harpadon nehereus* | Bombay-duck | NT | B, M | B | O | Near threatened |  | 12S |
|  |  | *Saurida undosquamis* | Brushtooth lizardfish | LC | M | R | A |  |  | 12S |
| Batrachoidiformes | Batrachoididae | *Batrachomoeus trispinosus* | Three-spined frogfish | NE | B, M | R |  |  | X | 12S |
| Beloniformes | Adrianichthyidae | *Oryzias melastigma* | Marine medaka | LC | F, B | B |  |  |  | 12S |
|  |  | **^Ƀ^***Oryzias javanicus* | Javanese ricefish | LC | F, B | BP |  |  | X | COI, 12S |
|  | Belonidae | *Ablennes hians* | Flat needlefish | LC | B, M | R | O |  |  | 12S |
|  |  | *Tylosurus crocodilus* | Hound needlefish | LC | M | R | O |  |  | COI, 12S |
|  | Exocoetidae | *Parexocoetus brachypterus* | Sailfin flyingfish | NE | M | N | O |  |  | 12S |
|  | Zenarchopteridae | *Zenarchopterus buffonis* | Buffon's river-garfish | NE | B, M | R |  |  | X | 12S |
|  |  | **Dermogenys collettei* | Halfbeak | LC | F, B, M | N |  | BLAST detection as *Dermogenys pusilla*. Refer to Note^4^. |  | 12S |
|  |  | *Zenarchopterus dunckeri* | Duncker's river garfish | NE | B | P |  |  |  | 12S |
| Blenniiformes | Blenniidae | *Omobranchus punctatus* | Muzzled blenny | LC | B, M | B |  |  |  | 12S |
| Carangiformes | Carangidae | *Atule mate* | Yellowtail scad | LC | B, M | R |  |  | X | 12S |
|  |  | *Scomberoides tol* | Needlescaled queenfish | LC | B, M | R |  |  | X | 12S |
|  |  | *Alepes vari* | Herring scad | LC | B, M | N |  |  |  | 12S |
|  |  | *Carangoides praeustus* | Brownback trevally | LC | M | D | O |  |  | 12S |
|  |  | *Decapterus macrosoma* | Shortfin scad | LC | M | R |  |  |  | 12S |
|  |  | *Selar crumenophthalmus* | Bigeye scad | LC | M | R |  |  |  | 12S |
|  |  | **^Ƀ^***Caranx ignobilis* | Giant trevally | LC | B, M | R |  |  | X | COI, 12S |
|  |  | **^Ƀ^***Megalaspis cordyla* | Torpedo scad | LC | M | R |  |  | X | COI |
|  |  | **^Ƀ^***Scomberoides commersonnianus* | Talang queenfish | LC | B, M | R | AM |  | X | COI |
|  |  | *Carangoides malabaricus* | Malabar trevally |  | M | R | A |  |  | COI, 12S |
|  |  | *Caranx melampygus* | Bluefin trevally | LC | B, M | R |  |  |  | COI, 12S |
|  |  | *Decapterus maruadsi* | Japanese scad | LC | M | R |  |  |  | COI, 12S |
|  |  | *Scomberoides lysan* | Doublespotted queenfish | LC | B, M | R |  |  |  | COI |
|  | Coryphaenidae | *Coryphaena hippurus* | Common dolphinfish | LC | B, M | N | O |  |  | 12S |
|  | Polynemidae | *Eleutheronema tetradactylum* | Fourfinger threadfin | NE | F, B, M | N | AM |  | X | 12S |
|  |  | *Polydactylus sextarius* | Blackspot threadfin | NE | B, M | D | A |  |  | 12S |
|  | Latidae | **^Ƀ^***Lates calcarifer* | Barramundi | LC | F, B, M | D | C |  | X | COI, 12S |
|  | Toxotidae | *Toxotes chatareus* | Spotted archerfish | LC | F, B | P | A |  |  | 12S |
|  | Cynoglossidae | *Cynoglossus bilineatus* | Fourlined tonguesole | LC | B, M | D |  |  | X | 12S |
|  |  | *Cynoglossus lingua* | Long tongue sole | LC | F, B, M | D | AM |  | X | 12S |
|  |  | *Cynoglossus puncticeps* | Speckled tonguesole | LC | F, B, M | D |  |  | X | 12S |
|  | Psettodidae | *Psettodes erumei* | Indian halibut | DD | M | D |  |  |  | 12S |
|  | Soleidae | *Solea ovata* | Ovate sole | LC | M | D |  |  |  | 12S |
|  |  | *Zebrias quagga* | Fringefin zebra sole | LC | M | D | A |  |  | 12S |
| Centrarchiformes | Cirrhitidae | *Oxycirrhites typus* | Longnose hawkfish | LC | M | R | NM |  |  | COI |
|  | Terapontidae | *Terapon jarbua* | Jarbua terapon | LC | F, B, M | D | C |  | X | 12S |
|  |  | *Terapon theraps* | Largescaled terapon | LC | F, B, M | R |  |  | X | 12S |
| Cichliformes | Cichlidae | *Oreochromis niloticus* | Nile tilapia | LC | F, B | B | P | Invasive |  | 12S |
|  |  | *Oreochromis* sp. 1 | Tilapia |  |  |  |  |  |  | 12S |
|  |  | *Oreochromis* sp. 2 | Tilapia |  |  |  |  |  |  | 12S |
| Clupeiformes | Chirocentridae | *Chirocentrus dorab* | Dorab wolf-herring | LC | B, M | R | A |  |  | 12S |
|  | Clupeidae | *Anodontostoma chacunda* | Chacunda gizzard shad | LC | F, B, M | N | AN |  | X | 12S |
|  |  | *Escualosa thoracata* | White sardine | LC | F, B, M | N | AM |  | X | 12S |
|  |  | *Amblygaster clupeoides* | Bleeker smoothbelly sardinella | LC | M | R |  |  |  | COI |
|  | Engraulidae | **Stolephorus* sp*.* | Anchovy |  |  |  |  | BLAST detection as *Stolephorus dubiosus*. Refer to Note^5^. |  | 12S |
|  |  | ********Encrasicholina heteroloba* | Shorthead anchovy | LC | M | R | O | BLAST detection as *Encrasicholina devisi*. Refer to Note^6^. |  | 12S |
|  |  | ********Encrasicholina pseudoheteroloba* | Shorthead anchovy | LC | M | R | O | BLAST detection as *Encrasicholina heteroloba*. Refer to Note^6^. |  | 12S |
|  |  | *Encrasicholina punctifer* | Buccaneer anchovy | LC | M | R | O |  |  | 12S |
|  |  | *Stolephorus baganensis* | Bagan anchovy | LC | B, M | N | A |  | X | 12S |
|  |  | *Thryssa baelama* | Baelama anchovy | LC | M | P |  |  |  | 12S |
|  |  | **^Ƀ^***Thryssa kammalensis* | Kammal thryssa | DD | B, M | N | O |  | X | COI |
|  | Pristigasteridae | **^Ƀ^***Ilisha melastoma* | Indian ilisha | LC | B, M | N | AM |  | X | COI |
| Cypriniformes | Cyprinidae | *Barbodes binotatus* | Spotted barb | LC | F | B |  |  |  | 12S |
|  |  | *Esomus metallicus* | Flying barb | LC | F, B | B |  |  |  | 12S |
|  |  | ********Mystacoleucus obtusirostris* | Masai barb | LC | F | B |  | BLAST detection as *Mystacoleucus marginatus*. Refer to Note^7^. |  | 12S |
|  |  | *Tor tambra* | Malayan mahseer | DD | F | B |  |  |  | 12S |
|  |  | *Rasbora pauciperforata* | Redstripe rasbora | LC | F | B |  |  |  | COI |
|  |  | *Tor* sp. | Mahseer | DD | F | B | P |  |  | COI |
| Cyprinodontiformes | Aplocheilidae | ********Aplocheilus armatus* | Blue panchax | LC | F, B | B |  | BLAST detection as *Aplocheilus panchax*. Refer to Note^8^. |  | 12S |
|  | Poeciliidae | *Poecilia vivipara* | Guppy | NE | F, B | B |  | Invasive |  | COI |
| Elopiformes | Elopidae | *Elops machnata* | Tenpounder | LC | B, M | N | O |  |  | 12S |
| Gobiiformes | Eleotridae | *Butis butis* | Duckbill sleeper | LC | F, B, M | D |  |  | X | 12S |
|  |  | *Butis koilomatodon* | Mud sleeper | NE | B, M | D | AM |  | X | 12S |
|  |  | *Butis melanostigma* | Black-spotted gudgeon | NE | F, B, M | D | A |  |  | 12S |
|  |  | *Butis* sp. | Gudgeon |  |  |  |  |  |  | 12S |
|  |  | ********Giuris margaritaceus* | Snakehead gudgeon | LC | F, B, M | D | A | BLAST detection as *Giuris margaritacea*. Refer to Note^9^. |  | 12S |
|  |  | *Oxyeleotris marmorata* | Marble goby | LC | F, B | D |  |  |  | 12S |
|  |  | *Ophiocara porocephala* | Northern mud gudgeon | LC | F, B, M | A |  |  |  | COI, 12S |
|  | Gobiidae | *Acentrogobius caninus* | Tropical sand goby | LC | B, M | D | AM |  | X | 12S |
|  |  | *Boleophthalmus boddarti* | Boddart's goggle-eyed goby | LC | F, B, M | D | AM |  | X | 12S |
|  |  | *Exyrias puntang* | Puntang goby | LC | B, M | R |  |  | X | 12S |
|  |  | *Pseudogobius fulvicaudus* | Oxudercid | NE | B | D |  |  | X | 12S |
|  |  | *Pseudogobius olorum* | Bluespot goby | NE | F, B, M | D |  |  | X | 12S |
|  |  | *Trypauchen vagina* | Mudburrowing goby | LC | B, M | D | AM |  | X | 12S |
|  |  | *Acentrogobius janthinopterus* | Robust mangrove goby | NE | F, B, M | R | A |  |  | 12S |
|  |  | *Asterropteryx semipunctata* | Starry goby | LC | M | R |  |  |  | 12S |
|  |  | *Boleophthalmus pectinirostris* | Great blue spotted mudskipper | NE | F, B, M | D |  |  |  | 12S |
|  |  | *Drombus triangularis* | Brown drombus | LC | F, B, M | D | A |  |  | 12S |
|  |  | ********Pseudogobiopsis oligactis* | Goby | LC | F, B | D |  | BLAST detection as *Eugnathogobius oligactis*. Refer to Note^10^. |  | 12S |
|  |  | *Eugnathogobius variegatus* | Gudgeon | LC | B, M | D |  |  |  | 12S |
|  |  | *Mugilogobius* sp. | Goby |  |  |  |  |  |  | 12S |
|  |  | *Periophthalmodon schlosseri* | Giant mudskipper | LC | F, B, M | D | A |  |  | 12S |
|  |  | ********Pseudogobius poicilosoma* | Northern fatnose goby | LC | F, B, M | B |  | BLAST detection as *Pseudogobius javanicus*. Refer to Note^11^. |  | 12S |
|  |  | *Scartelaos histophorus* | Walking goby | LC | B, M | D |  |  |  | 12S |
|  |  | **^Ƀ^***Hemigobius hoevenii* | Banded mulletgoby | NE | F, B, M | D | AM |  | X | COI, 12S |
|  |  | *Stigmatogobius pleurostigma* | Gudgeon | NE | F, B | B |  |  |  | COI |
| Gonorynchiformes | Chanidae | *Chanos chanos* | Milkfish | LC | F, B, M | B | A |  |  | COI, 12S |
| Holocentriformes | Holocentridae | *Sargocentron punctatissimum* | Speckled squirrelfish | LC | M | R |  |  |  | COI |
| Mugiliformes | Mugilidae | *Crenimugil crenilabis* | Fringelip mullet | LC | F, B, M | R | NM |  | X | 12S |
|  |  | *Ellochelon vaigiensis* | Squaretail mullet | LC | F, B, M | D | C |  | X | 12S |
|  |  | *Planiliza subviridis* | Greenback mullet | NE | F, B, M | D | C |  | X | 12S |
|  |  | *Planiliza macrolepis* | Largescale mullet | LC | F, B, M | D | C |  |  | 12S |
|  |  | *Mugil cephalus* | Flathead grey mullet | LC | F, B, M | B | C |  |  | 12S |
|  |  | *Paramugil parmatus* | Broad-mouthed mullet | NE | F, B, M | D | C |  |  | COI |
| Perciformes | Gerreidae | *Gerres filamentosus* | Whipfin silver-biddy | LC | F, B, M | D | AM |  | X | 12S |
|  | Apogonidae | *Ostorhinchus semilineatus* | Half-lined cardinal | DD | B, M | R |  |  |  | 12S |
|  | Labridae | *Calotomus spinidens* | Spinytooth parrotfish | LC | M | R |  |  |  | COI |
|  |  | *Pseudocheilinus hexataenia* | Sixline wrasse | LC | M | R |  |  |  | COI |
|  | Haemulidae | **^Ƀ^***Pomadasys kaakan* | Javelin grunter | NE | B, M | R |  |  | X | COI, 12S |
|  | Lutjanidae | *Lutjanus argentimaculatus* | Mangrove red snapper | LC | B, M | R | O |  | X | 12S |
|  |  | *Lutjanus decussatus* | Checkered snapper | LC | M | R |  |  |  | 12S |
|  |  | *Lutjanus rivulatus* | Blubberlip snapper | LC | M | R |  |  |  | 12S |
|  |  | **^Ƀ^***Lutjanus johnii* | John's snapper | LC | B, M | R | O |  | X | COI, 12S |
|  |  | *Lutjanus indicus* | Snapper | LC | F, B, M | R |  |  |  | COI |
|  |  | *Lutjanus malabaricus* | Malabar blood snapper | LC | B, M | R |  |  |  | COI, 12S |
|  | Ambassidae | *Ambassis vachellii* | Vachelli's glass perchlet | NE | F, B, M | D | O |  | X | 12S |
|  |  | *Ambassis nalua* | Scalloped perchlet | LC | F, B, M | D | A |  |  | COI |
|  | Platycephalidae | *Platycephalus indicus* | Bartail flathead | DD | B, M | R | O |  | X | 12S |
|  | Pomacentridae | *Abudefduf sordidus* | Blackspot sergeant | LC | B, M | R | NM |  |  | 12S |
|  |  | *Neoglyphidodon melas* | Bowtie damselfish | NE | M | R | NM |  |  | 12S |
|  | Sciaenidae | *Dendrophysa russelii* | Goatee croaker | LC | F, B, M | D | AM |  | X | 12S |
|  |  | *Johnius amblycephalus* | Bearded croaker | LC | F, B, M | D |  |  | X | 12S |
|  |  | *Johnius belangerii* | Belanger's croaker | LC | B, M | D | AM |  | X | 12S |
|  |  | *Pennahia anea* | Donkey croaker | LC | B, M | D |  |  | X | 12S |
|  |  | *Chrysochir aureus* | Reeve's croaker | LC | B, M | B |  |  |  | 12S |
|  |  | **^Ƀ^***Otolithes ruber* | Tigertooth croaker | LC | B, M | BP | AM |  | X | COI, 12S |
|  |  | **^Ƀ^***Pennahia ovata* | Croaker | DD | M | BP |  |  | X | COI |
|  |  | **^Ƀ^***Nibea soldado* | Soldier croaker | LC | F, B, M | D | A |  | X | COI |
|  | Scorpaenidae | *Pterois volitans* | Red lionfish | LC | M | R |  |  |  | COI |
|  | Serranidae | *Epinephelus bleekeri* | Duskytail grouper | DD | M | D |  |  | X | 12S |
|  |  | *Epinephelus coioides* | Orange-spotted grouper | LC | B, M | R |  |  | X | 12S |
|  |  | *Epinephelus sexfasciatus* | Sixbar grouper | LC | M | R |  |  | X | 12S |
|  |  | *Cephalopholis sonnerati* | Tomato hind | LC | M | R | NM |  |  | 12S |
|  |  | *Epinephelus chlorostigma* | Brownspotted grouper | LC | M | R | NM |  |  | 12S |
|  |  | *Epinephelus lanceolatus* | Giant grouper | DD | B, M | R |  |  |  | 12S |
|  |  | *Variola albimarginata* | White-edged lyretail | LC | M | R |  |  |  | 12S |
|  |  | *Epinephelus areolatus* | Areolate grouper | LC | M | R |  |  |  | COI |
|  |  | *Plectropomus laevis* | Blacksaddled coralgrouper | LC | M | R |  |  |  | COI |
|  | Sillaginidae | **^Ƀ^***Sillago sihama* | Silver sillago | LC | B, M | R | AM |  | X | COI, 12S |
|  | Sphyraenidae | *Sphyraena barracuda* | Great barracuda | LC | B, M | R |  |  | X | 12S |
|  |  | *Sphyraena qenie* | Blackfin barracuda | NE | M | R |  |  | X | 12S |
|  | Tetrarogidae | *Trichosomus trachinoides* | Waspfish | NE | M | D |  |  | X | 12S |
|  | Nemipteridae | *Nemipterus bipunctatus* | Delagoa threadfin bream | LC | M | D | NM |  |  | 12S |
|  |  | *Nemipterus marginatus* | Red filament threadfin bream | LC | M | D | NM |  |  | COI |
|  | Mullidae | *Parupeneus rubescens* | Rosy goatfish | LC | M | R |  |  |  | 12S |
|  |  | *Upeneus tragula* | Freckled goatfish | LC | B, M | R | O |  |  | 12S |
| Scombriformes | Gempylidae | *Rexea prometheoides* | Royal escolar | NE | M |  | B |  |  | COI |
|  | Scombridae | *Scomberomorus commerson* | Narrow-barred Spanish mackerel | NT | M | N | O | Near threatened |  | 12S |
|  |  | *Rastrelliger brachysoma* | Short mackerel | DD | B, M | N | O |  |  | COI, 12S |
|  |  | *Rastrelliger kanagurta* | Indian mackerel | DD | M | N | O |  |  | COI |
|  | Trichiuridae | *Lepturacanthus savala* | Savalai hairtail | NE | M, B | BP |  |  | X | 12S |
|  |  | *Trichiurus japonicus* | Largehead hairtail | LC | B, M | B | A |  |  | 12S |
| Siluriformes | Ariidae | *Hexanematichthys sagor* | Sagor catfish | NE | B, M | D | AM |  | X | 12S |
|  |  | *Plicofollis platystomus* | Flatmouth sea catfish | LC | B, M | D | AM |  | X | 12S |
|  |  | **^Ƀ^***Plicofollis argyropleuron* | Longsnouted catfish | NE | B, M | D |  |  | X | COI, 12S |
|  |  | *Arius jella* | Blackfin sea catfish | NE | B, M | D | A |  |  | COI |
|  |  | *Nemapteryx caelata* | Engraved catfish | NE | B, M | D | A |  |  | COI, 12S |
|  | Plotosidae | **^Ƀ^***Plotosus canius* | Gray eel-catfish | NE | F, B, M | D | AM |  | X | COI, 12S |
|  |  | *Plotosus lineatus* | Striped eel catfish | NE | B, M | R | A |  |  | COI, 12S |
| Syngnathiformes | Syngnathidae | *Hippichthys spicifer* | Bellybarred pipefish | NE | F, B, M | D |  |  |  | 12S |
| Tetraodontiformes | Tetraodontidae | *Dichotomyctere fluviatilis* | Green pufferfish | LC | F, B | D | P |  | X | 12S |
|  |  | **^Ƀ^***Dichotomyctere nigroviridis* | Spotted green pufferfish | NE | F, B | D |  |  | X | COI, 12S |
|  | Triacanthidae | *Triacanthus biaculeatus* | Short-nosed tripodfish | NE | B, M | D |  |  |  | 12S |
| **Number of species: 178**  **Number of genera: 127**  **Number of families: 68**  **Number of orders: 25**  **Number of classes: 2** | | | | | | | | | | |

**^a^**LC: Least Concern; VU: Vulnerable; NT: Near Threatened; EN: Endangered; NE: Not Evaluated; DD: Data Deficient

**^b^**M: Marine; B: Brackish; F: Freshwater

**^c^**D: Demersal; R: Reef-associated; BP: Benthopelagic; N: Neritic

**^d^**AM: Amphidromous; AN: Anadromous; C: Catadromous; O: Oceanodromous; P: Potamodromous; NM: Non-migratory

**^e^**Species overlapped with previous capture records^1,2,3^

**^Ƀ^**Species that have been COI-barcoded in ^1^.

**Note^1^** Changed *Leiognathus* cf. *ruconius* to *Deveximentum* sp.

**Reason:** The species “*ruconius*” is transferred to the genus “*Deveximentum*” (following^4^; although this taxonomic change is still disputed because there is no type specimen for this species). In addition, because *Deveximentum ruconius* is already listed and this list includes only distinct species, this species of *Deveximentum* must be an unidentified species.

**Note^2^** Changed *Channa gachua* to *Channa limbata*

**Reason:** Following the conclusions of species groupings and distribution by ^5^.

**Note^3^** Changed *Ophichthus evermanni* to *Ophichthus lithinus*

**Reason:** *Ophichthus evermanni* is currently considered a junior synonym of *Ophichthus* *lithinus* (se e^6^).

**Note^4^** Changed *Dermogenys pusilla* to *Dermogenys collettei*

**Reason:** All populations of *Dermogenys pusilla* of Peninsular Malaysia were described as a new species, *Dermogenys collettei*, by ^7^**.**

**Note^5^** Changed *Stolephorus dubiosus* to *Stolephorus* sp.

**Reason:** *Stolephorus dubiosus* is currently considered a junior synonym of *Stolephorus baganensis* (see ^8^) but *S. baganensis* is already listed; so, the reference sequence of “*Stolephorus dubiosus”* in GenBank must be from an “unidentified” species.

**Note^6^** Changed *Encrasicholina devisi* to *Encrasicholina heteroloba* and *Encrasicholina heteroloba* to *Encrasicholina pseudoheteroloba*

**Reason:** *Encrasicholina devisi* is currently considered a junior synonym of *Encrasicholina heteroloba* and *Encrasicholina pseudoheteroloba* was (until recently) misidentified as *Encrasicholina pseudoheteroloba* (see ^9^).

**Note^7^** Changed *Mystacoleucus marginatus* to *Mystacoleucus obtusirostris*

**Reason:** *Mystacoleucus marginatus* is currently considered a junior synonym of *Mystacoleucus obtusirostris* (see ^10^).

**Note^8^** Changed *Aplocheilus panchax* to *Aplocheilus armatus*

**Reason:** Following the taxonomic revision of ^11^ who recognized all population of *Aplocheilus* in Sundaland as *Aplocheilus armatus.*

**Note^9^** Changed *Giuris margaritacea* to *Giuris margaritaceus*

**Reason:** The gender of the genus name “*Giuris*” is masculine, therefore (according to ^10^) the species name must agree in gender with the genus name and be spelled “*margaritaceus*”.

**Note^10^** Changed *Eugnathogobius oligactis* to *Pseudogobiopsis oligactis*

**Reason:** following the reclassification of the species “*oligactis*” into the genus *Pseudogobiopsis*; see ^12^.

**Note^11^** Changed *Pseudogobius javanicus* to *Pseudogobius poicilosoma*

**Reason:** *Pseudogobius javanicus* is currently considered a junior synonym of *Pseudogobius poicilosoma* ^13^.

**Supplementary Table 3.** Fish capture records of Merbok Estuary based on previous species checklist (Mansor et al., 2012a; Zainal Abidin et al., 2021a, b). Where available, data on IUCN status, habitat, inhabited pelagic zone, and migration type of each species were compiled.

| Species (classified by order and family) | Common name | Reference^a^ | IUCN status^b^ | Habitat^c^ | Pelagic zone^d^ | Migration type^e^ |
| --- | --- | --- | --- | --- | --- | --- |
| MYLIOBATIFORMES |  |  |  |  |  |  |
| Dasyatidae Jordan & Gilbert 1879 |  |  |  |  |  |  |
| *Brevitrygon walga* (Müller & Henle 1841) | Scaly whipray | 1,2,3 | NT | M | D |  |
| *Telatrygon zugei* (Bürger 1841) | Pale-edged stingray | 2,3 | NT | M, B | D | AM |
| Gymnuridae Fowler 1934 |  |  |  |  |  |  |
| *Gymnura poecilura* (Shaw 1804) | Long-tailed butterfly ray | 1,2,3 | NT | M | D |  |
| ORECTOLOBIFORMES |  |  |  |  |  |  |
| Hemiscylliidae Gill 1862 |  |  |  |  |  |  |
| *Chiloscyllium indicum* (Gmelin 1789) | Slender bambooshark | 2,3 | NT | F, B, M | D | O |
| ANGUILLIFORMES |  |  |  |  |  |  |
| Ophichthidae Günther 1870 |  |  |  |  |  |  |
| *Pisodonophis cancrivorus* (Richardson 1848) | Longfin snake-eel | 2,3 | NE | F, B, M | R | AN |
| ELOPIFORMES |  |  |  |  |  |  |
| Elopidae Valenciennes 1847 |  |  |  |  |  |  |
| *Elops hawaiensis*Regan 1909 | Hawaiian ladyfish | 1 | DD | F, B, M | N | AN |
| *Megalops cyprinoides* (Broussonet 1782) | Indo-Pacific tarpon | 1 | DD | F, B, M | BP | AM |
| CLUPEIFORMES |  |  |  |  |  |  |
| Chirocentridae Bleeker 1849 |  |  |  |  |  |  |
| *Chirocentrus nudus* Swainson 1839 | Whitefin wolf-herring | 2,3 | LC | M | N |  |
| Clupeidae Cuvier 1816 |  |  |  |  |  |  |
| *Anodontostoma chacunda* (Hamilton 1822) | Chacunda gizzard shad | 1 | LC | F, B, M | N | AN |
| *Escualosa thoracata* (Valenciennes 1847) | White sardine | 2,3 | LC | F, B, M | N | AM |
| *Sardinella albella* (Valenciennes 1847) | White sardinella | 2,3 | LC | B, M | R |  |
| *Sardinella gibbosa* (Bleeker 1849) | Goldstripe sardinella | 2 | LC | M | N |  |
| Dussumieriidae Gill 1861 |  |  |  |  |  |  |
| *Dussumieria albulina* (Fowler 1934) | Lancer rainbow sardine | 2,3 | LC | M |  |  |
| Engraulidae Gill 1861 |  |  |  |  |  |  |
| *Setipinna taty* (Valenciennes 1848) | Scaly hairfin anchovy | 2,3 | LC | B, M | N | O |
| *Stolephorus baweanensis* Hardenberg 1933 | Hardenberg’s anchovy | 2,3 | DD | B, M | R | O |
| *Stolephorus mercurius* Hata, Lavoué and Motomura 2021 |  | 2,3 | NE | M |  |  |
| *Stolephorus baganensis* Hardenberg, 1933 | Bagan anchovy | 3 | LC | B, M | N | AM |
| *Stolephorus indicus* (van Hasselt 1823) | Indian anchovy | 2,3 | LC | B, M | N | O |
| *Stolephorus tri* (Bleeker 1852) | Spined anchovy | 1,2,3 | DD | B, M | N | AM |
| *Thryssa hamiltonii* (Gray 1835) | Hamilton's thryssa | 2,3 | LC | F, B, M | N | AM |
| *Thryssa kammalensis* (Bleeker 1849) | Kammal thryssa | 2,3 | DD | B, M | N | O |
| *Thryssa mystax* (Bloch & Schneider 1801) | Moustached thryssa | 2,3 | LC | B, M | P | O |
| *Encrasicholina punctifer* Fowler 1938 | Buccaneer anchovy | 1 | LC | M |  | O |
| Pristigasteridae Bleeker 1872 |  |  |  |  |  |  |
| *Ilisha melastoma* (Bloch & Schneider 1801) | Indian ilisha | 2,3 | LC | B, M | N | AM |
| *Opisthopterus tardoore* (Cuvier 1829) | Tardoore | 2,3 | LC | B, M | N | AM |
| SILURIFORMES |  |  |  |  |  |  |
| Ariidae Bleeker 1858 |  |  |  |  |  |  |
| *Arius gagora* (Hamilton 1822) | Gagora catfish | 2,3 | NT | F, B | D | AM |
| *Arius maculatus* (Thunberg 1792) | Spotted catfish | 1,2,3 | NE | B, M | D | P |
| *Arius caelatus* Valenciennes 1840 | Engraved catfish | 1 | NE | F, B, M | D | AM |
| *Plicofollis platystomus* (Day 1877) | Flatmouth sea catfish | 1 | LC | B, M | D | AM |
| *Hexanematichthys sagor* (Hamilton 1822) | Sagor catfish | 1,2,3 | NE | B, M | D | AM |
| *Ketengus typus* Bleeker 1846 | Bigmouth sea catfish | 2,3 | NE | F, B, M | D |  |
| *Osteogeneiosus militaris* (Linnaeus 1758) | Soldier catfish | 2,3 | NE | B, M | D |  |
| *Plicofollis argyropleuron* (Valenciennes 1840) | Longsnouted catfish | 1,2,3 | NE | B, M | D |  |
| *Plicofollis layardi* (Günther, 1866) | Thinspine sea catfish | 2,3 | NE | B, M | D | AM |
| *Plicofollis polystaphylodon* (Bleeker 1846) | Mozambique sea catfish | 2,3 | NE | F, B, M | D |  |
| Plotosidae Bleeker 1858 |  |  |  |  |  |  |
| *Plotosus canius* Hamilton 1822 | Gray eel-catfish | 1,2,3 | NE | F, B, M | D | AM |
| AULOPIFORMES |  |  |  |  |  |  |
| Synodontidae Gill 1861 |  |  |  |  |  |  |
| *Saurida micropectoralis* Shindo & Yamada 1972 | Shortfin lizardfish | 2,3 | LC | M | D |  |
| BATRACHOIDIFORMES |  |  |  |  |  |  |
| Batrachoididae Jordan 1896 |  |  |  |  |  |  |
| *Allenbatrachus grunniens* (Linnaeus 1758) | Grunting toadfish | 2,3 | NE | B, M | D | AM |
| *Batrachomoeus trispinosus* (Günther 1861) | Three-spined frogfish | 1,2,3 | NE | B, M | R |  |
| GOBIIFORMES |  |  |  |  |  |  |
| Eleotridae Bonaparte 1835 |  |  |  |  |  |  |
| *Butis butis* (Hamilton 1822) | Duckbill sleeper | 1,2,3 | LC | F, B, M | D |  |
| *Butis humeralis* (Valenciennes 1873) | Gudgeons | 2,3 | NE | B, M | D | AM |
| *Butis koilomatodon* (Bleeker 1849) | Mud sleeper | 2,3 | NE | B, M | D | AM |
| *Butis gymnopomus* (Bleeker 1853) | Goby | 1 | LC | F, B | D |  |
| Gobiidae Cuvier 1816 |  |  |  |  |  |  |
| *Acentrogobius caninus* (Valenciennes 1873) | Tropical sand goby | 2,3 | LC | B, M | D | AM |
| *Acentrogobius audax* Smith 1959 | Mangrove goby | 1 | LC | B, M | D |  |
| *Acentrogobius viridipunctatus* (Valenciennes 1837) | Spotted green goby | 1 | LC | B, M | D | AM |
| *Boleophthalmus pectinirostris* (Linnaeus 1758) | Great blue spotted mudskipper | 1 | NE | B, M | D |  |
| *Boleopthhalmus boddarti* (Pallas 1770) | Boddart’s goggle-eyed goby | 2,3 | LC | F, B, M | D | AM |
| *Brachygobius kabiliensis* Inger 1958 | Kabili bumblebee goby | 2,3 | LC | F, B, M | D |  |
| *Exyrias puntang* (Bleeker 1815) | Puntang goby | 2,3 | LC | B, M | R |  |
| *Favonigobius gymnauchen* (Bleeker 1860) | Sharp-nosed sand goby | 2,3 | NE | F, B, M | R | AM |
| *Glossogobius aureus* Akihito & Meguro 1975 | Golden tank goby | 2,3 | LC | F, B, M | D | AM |
| *Hemigobius hoevenii* (Bleeker 1851) | Banded mulletgoby | 2,3 | NE | F, B, M | D | AM |
| *Psammogobius biocellatus* (Valenciennes 1873) | Sleepy goby | 2,3 | LC | F, B, M | BP | AM |
| *Pseudapocryptes elongatus* (Cuvier 1816) | Mudskipper | 2,3 | LC | F, B, M | D | AM |
| *Pseudogobius fulvicaudus* Huang, Shao & Chen 2014 | Oxudercid | 2,3 | NE | B | D |  |
| *Pseudogobius avicennia*  (Herre 1940) | Goby | 3 | NE | F, B, M | BP |  |
| *Stigmatogobius sadanundio* (Hamilton 1822) | Oxudercid | 2,3 | NE | F, B, M | BP |  |
| *Trypauchen pelaeos*  Murdy 2006 | Mudburrowing goby | 2,3 | NE | B, M | D |  |
| *Trypauchen vagina*  (Bloch & Schneider 1801) | Mudburrowing goby | 2,3 | LC | B, M | D | AM |
| *Cryptocentrus* sp.  Valenciennes 1837 | Shrimp-goby | 3 |  |  |  |  |
| ATHERINIFORMES |  |  |  |  |  |  |
| Phallostethidae Regan 1916 |  |  |  |  |  |  |
| *Neostethus lankesteri* Regan 1916 | Priapum fish | 2,3 | NE | F, B | BP |  |
| BELONIFORMES |  |  |  |  |  |  |
| Adrianichthyidae Weber 1913 |  |  |  |  |  |  |
| *Oryzias javanicus* (Bleeker 1854) | Javanese ricefish | 2,3 | LC | F, B | BP |  |
| Belonidae Bonaparte 1835 |  |  |  |  |  |  |
| *Strongylura strongylura* (van Hasselt 1823) | Spottail needlefish | 1,2,3 | NE | B, M | N |  |
| Hemiramphidae Gill 1859 |  |  |  |  |  |  |
| *Hyporhamphus dussumieri* (Valenciennes 1847) | Dussumier's halfbeak | 2,3 | NE | M | R |  |
| *Hyporhamphus quoyi* (Valenciennes 1847) | Quoy’s garfish | 1,2,3 | NE | B, M | N |  |
| *Hemiramphus far* (Fabricius 1775) | Black-barred halfbeak | 1 | NE | B, M |  | NM |
| Zenarchopteridae Fowler 1934 |  |  |  |  |  |  |
| *Dermogenys sumatrana* (Bleeker 1854) | Halfbeak | 2,3 | NE | F, B | P |  |
| *Zenarchopterus* *buffonis* (Valenciennes 1847) | Buffon’s river-garfish | 2 | NE | B, M | R |  |
| CARANGIFORMES |  |  |  |  |  |  |
| Carangidae Rafinesque 1815 |  |  |  |  |  |  |
| *Alepes melanoptera* (Swainson 1839) | Blackfin scad | 2,3 | LC | B, M | N |  |
| *Atule mate* (Cuvier 1833) | Yellowtail scad | 2,3 | LC | B, M | R |  |
| *Carangoides coeruleopinnatus* (Rüppell 1830) | Coastal trevally | 2,3 | LC | M | R |  |
| *Carangoides praeustus* (Anonymous [Bennett] 1830) | Brownback trevally | 1 | LC | B, M | D | O |
| *Carangoides talamporoides* Bleeker, 1852 | Imposter trevally | 1 | LC | M | D | O |
| *Carangoides uii* (Wakiya 1924) | Coastal trevally | 1 | LC | M | D |  |
| *Caranx ignobilis* (Forsskål 1775) | Giant trevally | 2,3 | LC | B, M | R |  |
| *Caranx sexfasciatus* Quoy & Gaimard 1825 | Bigeye trevally | 1,2,3 | LC | B, M | R | AM |
| *Megalaspis cordyla* (Linnaeus 1758) | Torpedo scad | 2,3 | LC | M | R |  |
| *Scomberoides commersonnianus* Lacepède 1801 | Talang queenfish | 2,3 | LC | B, M | R | AM |
| *Scomberoides tala* (Cuvier 1832) | Barred queenfish | 2,3 | LC | M | R |  |
| *Scomberoides tol* (Cuvier 1832) | Needlescaled queenfish | 2,3 | LC | B, M | R |  |
| *Selaroides leptolepis* (Cuvier 1833) | Yellowstripe scad | 2,3 | LC | F, B, M | R | AM |
| *Trachinotus blochii* (Lacepède 1801) | Snubnose pompano | 2,3 | LC | B, M | R |  |
| *Ulua mentalis* (Cuvier 1833) | Longrakered trevally | 2,3 | LC | M | R |  |
| Cynoglossidae Jordan 1888 |  |  |  |  |  |  |
| *Cynoglossus arel* (Bloch & Schneider 1801) | Largescale tonguesole | 2,3 | DD | F, B, M | D |  |
| *Cynoglossus* cf. *cynoglossus* | Tonguesole | 3 |  |  |  |  |
| *Cynoglossus monopus* (Bleeker, 1849) | Tonguesole | 3 | LC | M | D |  |
| *Cynoglossus oligolepis* (Bleeker 1855) |  | 2,3 | NE | M | D |  |
| *Cynoglossus bilineatus* (Lacepède, 1802) | Fourlined tonguesole | 1,2,3 | LC | B, M | D |  |
| Bothidae Smitt 1892 |  |  |  |  |  |  |
| *Grammatobothus polyophthalmus* (Bleeker, 1865) | Threespot flounder | 1 | LC | M | D |  |
| Paralichthyidae Regan 1910 |  |  |  |  |  |  |
| *Pseudorhombus arsius*  (Hamilton, 1822) | Largetooth flounder | 3 | NE | B, M | D | O |
| Latidae Jordan 1888 |  |  |  |  |  |  |
| *Lates calcarifer* (Bloch, 1790) | Barramundi | 1,2,3 | LC | F, B, M | D | C |
| Polynemidae Rafinesque 1815 |  |  |  |  |  |  |
| *Eleutheronema tetradactylum* (Shaw 1804) | Fourfinger threadfin | 1,2,3 | NE | F, B, M | N | AM |
| *Leptomelanosoma indicum* (Shaw 1804) | Indian threadfin | 2,3 | NE | B, M | D | AM |
| MUGILIFORMES |  |  |  |  |  |  |
| Mugilidae Jarocki 1822 |  |  |  |  |  |  |
| *Crenimugil buchanani* (Bleeker 1853) | Bluetail mullet | 1,2,3 | LC | F, B, M | N | C |
| *Crenimugil crenilabis* (Forsskål 1775) | Fringelip mullet | 2,3 | LC | F, B, M | R | NM |
| *Osteomugil perusii* (Valenciennes 1836) | Longfinned mullet | 2,3 | LC | M | R |  |
| *Osteomugil engeli* (Bleeker 1858) | Kanda mullet | 1 | LC | F, B, M | D | C |
| *Planiliza subviridis* (Valenciennes 1836) | Greenback mullet | 1,2,3 | NE | F, B, M | D | C |
| *Planiliza tade* (Fabricius 1775) | Tade gray mullet | 1 | NE | F, B, M | D | C |
| *Ellochelon vaigiensis*(Quoy & Gaimard 1825) | Squaretail mullet | 1 | LC | F, B, M | D | C |
| *Osteomugil speigleri* (Bleeker 1858) | Speigler's mullet | 1 | NE | F, B, M | D | C |
| PERCIFORMES |  |  |  |  |  |  |
| Gerreidae Bleeker 1859 |  |  |  |  |  |  |
| *Gerres macracanthus* Bleeker 1854 | Longspine silverbiddy | 2 | NE | B, M | D |  |
| *Gerres filamentosus* Cuvier 1829 | Whipfin silver-biddy | 1,2,3 | LC | F, B, M | D | AM |
| *Gerres limbatus* Cuvier 1830 | Saddleback silver-biddy | 2,3 | LC | F, B, M | D | AM |
| *Gerres oyena* (Forsskål 1775) | Common silver-biddy | 1,2,3 | LC | B, M | R |  |
| *Gerres kapas* (Forsskål, 1775) | Common silver-biddy | 1 | LC | B, M |  |  |
| *Pentaprion longimanus* (Cantor 1849) | Longfin mojarra |  | LC | B, M | D |  |
| Ambassidae Klunzinger 1870 |  |  |  |  |  |  |
| *Ambassis vachellii* Richardson 1846 | Vachelli’s glass perchlet | 2,3 | NE | F, B, M | D | O |
| *Ambassis interrupta* Bleeker 1853 | Long-spined glass perchlet | 2,3 | NE | F, B, M | D | AM |
| *Ambassis macracanthus* Bleeker 1849 | Estuarine glass perchlet | 2,3 | DD | F, B | D |  |
| Haemulidae Gill 1885 |  |  |  |  |  |  |
| *Pomadasys kaakan* (Cuvier 1830) | Javelin grunter | 1,2,3 | NE | B, M | R |  |
| Lethrinidae Bonaparte 1831 |  |  |  |  |  |  |
| *Lethrinus lentjan* (Lacepède 1802) | Pink ear emperor | 1,2,3 | LC | B, M | R | NM |
| Lutjanidae Gill 1861 |  |  |  |  |  |  |
| *Lutjanus argentimaculatus* (Forsskål, 1775) | Mangrove red snapper | 1,2,3 | LC | B, M | R | O |
| *Lutjanus johnii* (Bloch 1792) | John's snapper | 1,2,3 | LC | B, M | R | O |
| *Lutjanus russellii* (Bleeker 1849) | Russell's snapper | 1,2,3 | LC | B, M | R |  |
| Sciaenidae Cuvier 1829 |  |  |  |  |  |  |
| *Dendrophysa russelii* (Cuvier 1829) | Goatee croaker | 1,2,3 | LC | F, B, M | D | AM |
| *Johnius belangerii* (Cuvier 1830) | Belanger's croaker | 1,2 | LC | B, M | D | AM |
| *Johnius* sp. | Croacker | 2,3 |  |  |  |  |
| *Johnius amblycephalus* (Bleeker 1855) | Bearded croaker | 1 | LC | F, B, M | D |  |
| *Johnius borneensis* (Bleeker 1851) | Sharpnose hammer croaker | 1 | LC | F, B, M | BP |  |
| *Paranibea semiluctuosa*(Cuvier 1830) | Half-mourning croaker | 1 | LC | M | D |  |
| *Otolithes ruber* (Bloch & Schneider 1801) | Tigertooth croaker | 2,3 | LC | B, M | BP | AM |
| *Panna microdon* (Bleeker 1849) | Panna croaker | 2,3 | LC | B, M | D |  |
| *Pennahia anea* (Bloch 1793) | Donkey croaker | 2,3 | LC | B, M | D |  |
| *Pennahia ovata* Sasaki 1996 | Croaker | 2,3 | DD | M | BP |  |
| Serranidae Swainson 1839 |  |  |  |  |  |  |
| *Cephalopholis formosa* (Shaw 1812) | Bluelined hind | 2,3 | LC | M | R | NM |
| *Epinephelus bleekeri* (Vaillant 1878) | Duskytail grouper | 2,3 | DD | M | D |  |
| *Epinephelus coioides* (Hamilton 1822) | Orange-spotted grouper | 1,2,3 | LC | B, M | R |  |
| *Epinephelus heniochus* Fowler 1904 | Bridled grouper | 2,3 | LC | M | D | NM |
| *Epinephelus sexfasciatus* (Valenciennes 1828) | Sixbar grouper | 2,3 | LC | M | R |  |
| Sillaginidae Richardson 1846 |  |  |  |  |  |  |
| *Sillago sihama* (Forsskål 1775) | Silver sillago | 1,2,3 | LC | B, M | R | AM |
| Sphyraenidae Rafinesque 1815 |  |  |  |  |  |  |
| *Sphyraena barracuda* (Edwards 1771) | Great barracuda | 1,2,3 | LC | B, M | R |  |
| *Sphyraena jello* Cuvier 1829 | Pickhandle barracuda | 1,2,3 | NE | M | R | O |
| *Sphyraena qenie* Klunzinger 1870 | Blackfin barracuda | 2,3 | NE | M | R |  |
| Platycephalidae Swainson 1839 |  |  |  |  |  |  |
| *Grammoplites scaber* (Linnaeus 1758) | Rough flathead | 2,3 | NE | B, M | D | AM |
| *Platycephalus indicus* (Linnaeus 1758) | Bartail flathead | 2,3 | DD | B, M | R | O |
| Tetrarogidae Smith 1949 |  |  |  |  |  |  |
| *Trichosomus trachinoides* (Cuvier 1829) | Waspfish | 2,3 | NE | M | D |  |
| CICHLIFORMES |  |  |  |  |  |  |
| Cichlidae Bonaparte 1835 |  |  |  |  |  |  |
| *Oreochromis mossambicus* (Peters 1852) | Mozambique tilapia | 1,2,3 | VU | F, B | BP | AM |
| ACANTHURIFORMES |  |  |  |  |  |  |
| Drepaneidae Gill 1872 |  |  |  |  |  |  |
| *Drepane longimana* (Bloch & Schneider 1801) | Concertina fish | 2 | NE | M | R | AM |
| *Drepane punctata* (Linnaeus 1758) | Spotted sicklefish | 2,3 | NE | B, M | R | AM |
| Leiognathidae Gill 1893 |  |  |  |  |  |  |
| *Deveximentum ruconius* (Hamilton 1822) | Deep pugnose ponyfish | 2,3 | NE | F, B, M | D | AM |
| *Deveximentum indicium* (Monkolprasit 1973) | Ponyfish | 2,3 | NE | B, M | N |  |
| *Deveximentum hanedai* (Mochizuki & Hayashi 1989) | Ponyfish | 2,3 | LC | B, M | D |  |
| *Eubleekeria jonesi* (James 1971) | Jones’ pony fish | 2,3 | NE | B, M | D |  |
| *Leiognathus brevirostris* (Valenciennes 1835) | Shortnose ponyfish | 2,3 | NE | B, M | D | AM |
| *Leiognathus equula* (Forsskål 1775) | Common ponyfish | 2,3 | LC | F, B | D | AM |
| *Nuchequula gerreoides* (Bleeker 1851) | Decorated ponyfish | 2,3 | NE | M | D | AM |
| *Nuchequula nuchalis* (Temminck & Schlegel 1845) | Spotnape ponyfish | 1 | NE | M, B | N |  |
| *Leiognathus smithursti*(Ramsay & Ogilby 1886) | Longspine ponyfish | 1 | NE | M | D |  |
| Scatophagidae Gill 1883 |  |  |  |  |  |  |
| *Scatophagus argus* (Linnaeus 1766) | Spotted scat | 1,2,3 | LC | F, B, M | R | AM |
| Siganidae Richardson 1837 |  |  |  |  |  |  |
| *Siganus fuscescens* (Houttuyn 1782) | Mottled spinefoot | 2,3 | LC | M | R | O |
| *Siganus javus* (Linnaeus 1766) | Streaked spinefoot | 1,2,3 | LC | B, M | R | O |
| *Siganus canaliculatus*(Park 1797) | White-spotted spinefoot | 1 | LC | M, B | R | O |
| *Siganus guttatus* (Bloch 1787) | Orange-spotted spinefoot | 1 | LC | M, B | R |  |
| SCOMBRIFORMES |  |  |  |  |  |  |
| Stromateidae Rafinesque 1810 |  |  |  |  |  |  |
| *Pampus argenteus* (Euphrasen 1788) | Silver pomfret | 1,2,3 | NE | M | BP | O |
| Trichiuridae Rafinesque 1810 |  |  |  |  |  |  |
| *Lepturacanthus savala* (Cuvier 1829) | Savalai hairtail | 2,3 | NE | M, B | BP |  |
| CENTRARCHIFORMES |  |  |  |  |  |  |
| Terapontidae Richardson 1842 |  |  |  |  |  |  |
| *Terapon jarbua* (Forsskål 1775) | Jarbua terapon | 1,2,3 | LC | F, B, M | D | C |
| *Terapon theraps* Cuvier 1829 | Largescaled terapon | 2,3 | LC | F, B, M | R |  |
| TETRAODONTIFORMES |  |  |  |  |  |  |
| Tetraodontidae Bonaparte 1831 |  |  |  |  |  |  |
| *Arothron reticularis* (Bloch & Schneider 1801) | Reticulated pufferfish | 2,3 | LC | B, M | R |  |
| *Dichotomyctere* cf. *fluviatilis* (Hamilton 1822) | Green pufferfish | 1,2,3 | LC | F, B | D | P |
| *Dichotomyctere nigroviridis* (Marion de Procé 1822) | Spotted green pufferfish | 1,2,3 | NE | F, B | D |  |
| *Lagocephalus lunaris* (Bloch & Schneider 1801) | Lunartail puffer | 2,3 | LC | B, M | D | O |
| *Takifugu oblongus* (Bloch 1786) | Lattice blaasop | 2,3 | LC | B, M | D |  |
| Triacanthidae Bleeker 1859 |  |  |  |  |  |  |
| *Triacanthus nieuhofii* Bleeker 1852 | Silver tripodfish | 2,3 | NE | M | D |  |
| Number of species: 165  Number of genera: 102  Number of families: 49  Number of orders: 19 | | | | | | |

**^a^**1: Mansor et al. (2012a); 2: Zainal Abidin et al., 2021a; 3: Zainal Abidin et al., 2021b

**^b^**LC: Least Concern; VU: Vulnerable; NT: Near Threatened; NE: Not Evaluated; DD: Data Deficient

**^c^**M: Marine; B: Brackish; F: Freshwater

**^d^**D: Demersal; R: Reef-associated; BP: Benthopelagic; N: Neritic

**^e^**AM: Amphidromous; AN: Anadromous; C: Catadromous; O: Oceanodromous; P: Potamodromous; NM: Non-migratory

**Supplementary Table 4:** Summary of sequencing read statistics for both COI and 12S metabarcoding assays.

| Metabarcoding assays | Raw reads | Filtered reads | Mean reads/sample | S.D. | Fish reads (%) | Total MOTUs | Fish MOTUs |
| --- | --- | --- | --- | --- | --- | --- | --- |
| COI | 12,958,643 | 11,041,004 | 184,016 | 29,217 | 3.90 | 8332 | 244 |
| 12S | 10,757,026 | 8,266,965 | 140,118 | 19,238 | 98.51 | 859 | 309 |

**S.D.**: Standard deviation

**References**

1. Zainal Abidin, D. H. *et al.* DNA-based taxonomy of a mangrove-associated community of fishes in Southeast Asia. *Scientific Reports* **11**, 1-15, doi:10.1038/s41598-021-97324-1 (2021).

2. Zainal Abidin, D. H. *et al.* Ichthyofauna of Sungai Merbok Mangrove Forest Reserve, northwest Peninsular Malaysia, and its adjacent marine waters. *Check List* **17**, 601 - 631, doi:10.15560/17.2.601 (2021).

3. Mansor, M., Mohammad-Zafrizal, M., Nur-Fadhilah, M., Khairun, Y. & Wan-Maznah, W. Temporal and spatial variations in fish assemblage structures in relation to the physicochemical parameters of the Merbok estuary, Kedah. *Journal of Natural Sciences Research* **2**, 110-127 (2012).

4. Chakrabarty, P., Amarasinghe, T. & Sparks, J. Rediscription of ponyfishes (Teleostei: Leiognathidae) of Sri Lanka and the status of Aurigequula Fowler 1918. *Ceylon Journal of Science (Biological Sciences)* **37** (2009).

5. Rüber, L., Tan, H. H. & Britz, R. Snakehead (Teleostei: Channidae) diversity and the Eastern Himalaya biodiversity hotspot. *Journal of Zoological Systematics and Evolutionary Research* **58**, 356-386 (2020).

6. McCosker, J. E. & Psomadakis, P. N. Snake eels of the genus Ophichthus (Anguilliformes: Ophichthidae) from Myanmar (Indian Ocean) with the description of two new species. *Zootaxa* **4526**, 71-83 (2018).

7. Meisner, A. D. Phylogenetic systematics of the viviparous halfbeak genera Dermogenys and Nomorhamphus (Teleostei: Hemiramphidae: Zenarchopterinae). *Zoological Journal of the Linnean Society* **133**, 199-283 (2001).

8. Hata, H., Lavoué, S. & Motomura, H. Taxonomic status of seven nominal species of the anchovy genus *Stolephorus* described by Delsman (1931), Hardenberg (1933), and Dutt and Babu Rao (1959), with redescriptions of *Stolephorus tri* (Bleeker 1852) and *Stolephorus waitei* Jordan and Seale 1926 (Clupeiformes: Engraulidae). *Ichthyological Research* **67**, 7-38, doi:https://doi.org/10.1007/s10228-019-00697-7 (2020).

9. Hata, H. & Motomura, H. Validity of *Encrasicholina pseudoheteroloba* (Hardenberg 1933) and redescription of *Encrasicholina heteroloba* (Rüppell 1837), a senior synonym of *Encrasicholina devisi* (Whitley 1940)(Clupeiformes: Engraulidae). *Ichthyological Research* **64**, 18-28 (2017).

10. Kottelat, M. The fishes of the inland waters of Southeast Asia: a catalogue and core bibliography of the fishes known to occur in freshwaters, mangroves and estuaries. *Raffles Bulletin of Zoology* **27**, 1-663 (2013).

11. Katwate, U., Kumkar, P., Britz, R., Raghavan, R. & Dahanukar, N. The identity of *Aplocheilus andamanicus* (Köhler, 1906)(Teleostei: Cyprinodontiformes), an endemic Killifish from the Andaman Islands, with notes on *Odontopsis armata* van Hasselt. *Zootaxa* **4382**, 159-174 (2018).

12. Larson, H., Hadiaty, R. & Hubert, N. A new species of the gobiid fish genus *Pseudogobiopsis* (Teleostei, Gobiidae, Gobionellinae) from Indonesia. *The Raffles Bulletin of Zoology* **65**, 175-180 (2017).

13. Larson, H. K. & Hammer, M. P. A revision of the gobiid fish genus *Pseudogobius* (Teleostei, Gobiidae, Tridentigerinae), with description of seven new species from Australia and South-east Asia. *Zootaxa* **4961**, 1–85-81–85 (2021).
